# A Beginner's How‐To Guide to Urban Population Genetics and Genomics

**DOI:** 10.1002/ece3.73372

**Published:** 2026-04-07

**Authors:** Elizabeth J. Carlen, Lindsay S. Miles, Kevin Aviles‐Rodriguez, Warren Booth

**Affiliations:** ^1^ Living Earth Collaborative Washington University in St. Louis St. Louis Missouri USA; ^2^ Department of Entomology Virginia Polytechnic Institute and State University Blacksburg Virginia USA; ^3^ Johnson and Wales University Providence Rhode Island USA

**Keywords:** adaptation, conservation, evolution, genetic diversity, landscape connectivity, management, wildlife

## Abstract

Urbanization is one of the most significant drivers of environmental change, shaping the ecological and evolutionary processes of plants and animals. Understanding how species evolve in urban landscapes requires integrating population genetics and genomics with urban ecology. Thus, accessible guidance is necessary to facilitate interdisciplinary approaches for applying population genetic and genomic tools to understand the ecology and evolution of urban species. Here, we present a how‐to guide with key concepts and methodologies for studying urban population genetics, including identifying genetic markers, choosing appropriate analytical tools, and applying spatial genetic modeling approaches. We emphasize practical applications to assess genetic diversity, population connectivity, and adaptation, relevant in the generation of management strategies for conservation, pest control, and assisted gene flow. By bridging the gap between population genomics and urban ecology, this guide aims to equip researchers, wildlife managers, and conservation practitioners with essential tools to study and manage urban populations. Strengthening collaborations between urban ecologists, pest management professionals, geneticists, and city planners will enhance our ability to develop sustainable cities that support biodiversity, mitigate urban pests and invasive species, and promote coexistence between urban development and nature.

## Graphical abstract

This guide provides practical methodologies for studying how urbanization drives evolutionary and ecological changes in species through population genetics and genomics, covering genetic markers, analytical tools, and spatial modeling approaches. The framework enables researchers and practitioners to assess genetic diversity, connectivity, and adaptation to inform conservation strategies, pest control, and urban biodiversity management.

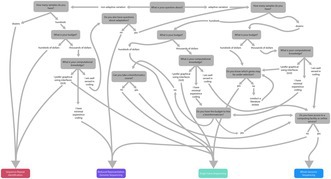

## 1 Introduction

Urban areas are among the fastest growing ecosystems (United Nations 2014) and can drastically alter the landscapes upon which they are built (McKinney 2006; Grimm et al. 2008). These alterations of the habitat (e.g., road networks, increased temperatures and pollution, reduced green spaces) can have drastic ecological and evolutionary consequences for species living in and near cities (Johnson and Munshi‐South 2017). While urban ecology has advanced our understanding of how anthropogenic landscapes function (Magle et al. 2012; Collins et al. 2021), less is known about how organisms are evolving in response to these environments (Sih et al. 2011). Population genetics/genomics provides a powerful framework for addressing this gap by revealing the evolutionary dynamics of urban‐associated species (Supple and Shapiro 2018; Luikart et al. 2019; Rajora 2024), including how they adapt, evolve, and disperse in human‐modified ecosystems (Hohenlohe et al. 2021). As we gain understanding of how cities can influence the evolutionary trajectories of populations, we can apply this knowledge to better inform management strategies and develop urban planning guidelines that take into consideration the needs of urban organisms.

Cities are complex systems shaped by factors such as age, culture, religion, politics, and geography (Carlen et al. 2025). The impact of urbanization on organisms depends heavily on their natural history and life cycle, necessitating a foundational understanding of both the species and the urban landscape when applying a genomic framework. For instance, pigeons ( *Columba livia* ) in the Northeastern United States can disperse over 1000 km, forming a single population across adjacent cities (Carlen and Munshi‐South 2021), whereas white‐footed mice ( *Peromyscus leucopus* ), with limited vagility (2–3 km) and canopy fragmentation, are confined to individual parks in New York City (Munshi‐South and Kharchenko 2010; Munshi‐South 2012). Synanthropic pests like German cockroaches ( *Blattella germanica* ) and bed bugs ( *Cimex lectularius* ) exhibit genetic structure influenced by human movement and building connectivity (Crissman et al. 2010; Booth et al. 2012; Saenz et al. 2012; Booth 2024; Tang et al. 2024; Miles, Verrelli, et al. 2025). Urban history also plays a critical role: city walls in Oviedo, Spain, isolated fire salamander ( *Salamandra salamandra* ) populations (Lourenço et al. 2017; IUCN SSC Amphibian Specialist Group 2023), while brown rats ( *Rattus norvegicus* ) reflect European colonization patterns due to ship‐borne dispersal (Puckett et al. 2016). Thus, integrating species biology with urban context is essential in urban population genomics.

Habitat loss due to human development is also of significant concern as it is a major driver of global biodiversity loss (Sih et al. 2000). In response, international treaties are incentivizing the restoration of degraded habitats (see United Nations 2019), which may include reintroducing lost species or bolstering populations of those in decline. Population genomics offers a quantitative framework to assess the effectiveness of restoration efforts. For example, Wright et al. (2022) used reduced representation sequencing on the common native bush rat, *Rattus fuscipes* , to assess the genetic impacts of a reintroduction project aimed at habitat restoration in an urban reserve in Sydney, Australia. Genetic diversity was estimated prior to reintroduction and then 3 years post to determine the evolutionary response to re‐introduction of an admixed population. While genetic diversity had declined over the 3 year period, this was not significant; thus, reintroduction was deemed a success. Additionally, Flores‐Manzanero et al. (2022) used microsatellite markers to identify population reductions and structure due to habitat fragmentation in two critically endangered carnivores on the rapidly urbanizing tourist island of Cozumel, Mexico. Flores‐Manzanero et al. (2022) documented low effective population sizes (Box 3) in both species and genetic clustering. The authors suggest a comprehensive management plan that includes limiting interaction with humans, preserving non‐urban habitat, and increasing corridors with native vegetation. As highlighted, population genomics represents a powerful tool to assess the success and failure of restoration in light of urbanization.

**FIGURE 1:**
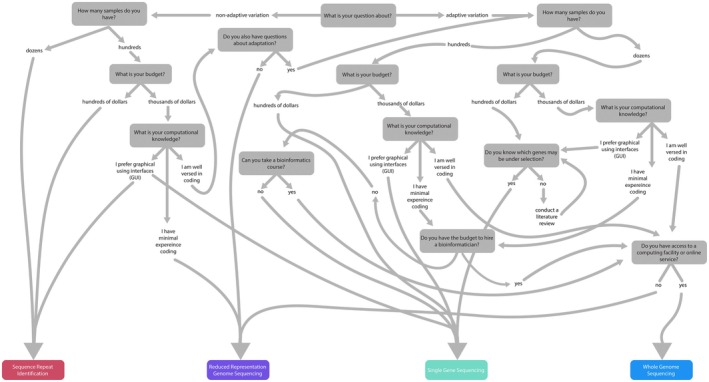
Decision tree to guide readers through the types of genetic sequencing that can be used to conduct population genetic studies based on the questions being asked, budget, and computational knowledge.

While genomic tools have shown to be invaluable in urban ecosystem assessment and management and the budding field of urban evolution, obstacles remain including engaging interdisciplinary researchers in the use of genetic tools for management and conservation decisions. Here, we show readers different scenarios and the considerations that are needed when tackling these questions (Figure 1). We provide multiple examples to introduce readers to the broad range of methods and taxa used to study urban management, conservation, and evolution. We aim to guide agencies responsible for wildlife management in urban centers, as well as early career urban ecologists and evolutionary biologists, with a “how‐to” guide to illustrate how population genetics may further our understanding of the evolutionary dynamics of urban species, which may better inform management strategies.

## 2 Marker Selection and Sequencing Techniques

All genetic studies require the selection of appropriate markers and sequencing techniques (Boxes 1 and 2). Choosing which markers are best suited will depend on the organism being studied, the question being asked, research budget, potential sample size, computational power, and bioinformatic proficiency (Figure 1). First, marker selection may be dependent on the type of organism being studied. For example, many plants are polyploid (i.e., anything greater than diploid) in their nuclear genome, thus polyploid organisms can be difficult to run population genomic analyses on as many of the software programs available assume haploid or diploid genomes. Second, it is essential to consider what questions are being asked about urban wildlife populations. For example, studies considering how urbanization influences population genetics across the geographic distribution of a species (e.g., broad range sampling) will have different constraints than studies examining fine‐scale population genetic signatures of urbanization. Additionally, if the goal of the research is to examine both adaptive (natural and sexual selection) and non‐adaptive evolution (genetic drift and gene flow), this should be considered at the onset of study design as not all DNA sequencing techniques are suitable for this work. Third, budget can have a huge impact on the suitability of sequencing techniques with platforms such as sequence repeat identification (Box 2) and reduced representation genome sequencing (Box 2) costing considerably less than other methods. Fourth, when sample size is limited, it is helpful to have more genetic markers. For example, Nazareno et al. (2017) found that only eight samples were necessary when at least 1000 Single Nucleotide Polymorphisms (SNPs) (Box 1) were used to estimate genetic diversity. In contrast, significant deviations in population genetic results were found when sample sizes were less than 30 when using 6 to 8 microsatellite loci (Reiner et al. 2019). Finally, computation power and bioinformatic knowledge should be considered as the data generated through different sequencing methods necessitate expertise in distinct computing platforms.

BOX 1 **Key genetic terms and makers.**

**Table:** 

*Allele*—one of two or more variants of a gene that arise via mutation and are found in the same place on a chromosome. *Genetic marker*—specific DNA sequence or gene that can be used to identify genetic variation, individuals, or species. *Locus*—the physical location of a gene or genetic marker on a chromosome; plural: loci. *Microsatellites (simple sequence repeats, SSRs)*—commonly used population genetic markers consisting of 2–4 base pair tandem repeats with high mutation rates (10^2^–10^6^ per cell generation). *Single nucleotide polymorphism (SNP)*—commonly used population genetic marker consisting of a nucleotide (adenine, thymine, cytosine, or guanine) that is different from the reference sequence. Here, SNPs are identified when using the sequencing of single genes, reduced representation genome sequencing and whole genome sequencing (see Box 2).

BOX 2 **Sequencing techniques commonly used in urban genetic studies.**

**Table:** 

*Single gene region sequencing* is a form of DNA sequencing in which known PCR and/or sequencing primers are used to obtain SNP data from a region of the genome (Palumbi et al. 2002). For population structure studies this is typically mitochondrial or chloroplast genes/intergenic regions, while for adaptation studies mitochondrial, chloroplast, or nuclear genes can be used (Freeland 2005). *Sequence repeat identification* is a method in which satellite DNA (DNA sequences that are organized as large arrays of tandemly repeated, short sequences), most commonly microsatellites due to their high mutation rates, are used to study recent evolutionary timescales. Because microsatellites are Mendelian bi‐parentally inherited, codominant, and often exhibit high levels of variation, they have been widely used to address questions ranging from pedigree and parentage inference to the identification of population structure (Avise 2012). For over 25 years (1989–2015), microsatellites were the predominant marker used in population genetic research (Vieira et al. 2016). However, in recent years, with the rise of next generation sequencing platforms, their use has decreased in favor of SNPs (Allendorf 2025). When microsatellite markers are available for a species of interest, or a closely related species, the cost to sequence microsatellite loci is low (Puckett 2016), particularly when loci can be multiplexed (i.e., multiple loci combined into a single sequencing reaction). If microsatellite markers have not been previously isolated, the cost of developing novel markers can be cost‐prohibitive (Guichoux et al. 2011; Antunes et al. 2022). That said, while classical methods used for the isolation of microsatellite loci involved cloning and enrichment for simple sequence repeats (Andrés and Bogdanowicz 2011), a time‐consuming procedure that often yielded low numbers of loci, next generation approaches such as reduced representation genome sequencing and low coverage whole genome sequencing, make it possible to rapidly identify thousands of loci for microsatellite development (Castoe et al. 2012). Regardless of which method is used to isolate microsatellite loci, locus optimization (i.e., determination of ideal polymerase chain reaction profile, primer and MgCl_2_ concentration, etc.) is required which may be laborious (Zane et al. 2002). Furthermore, not all microsatellite loci identified will prove suitable (e.g., lack of polymorphism, linkage disequilibrium with other markers identified, etc.). *Reduced representation genome sequencing* is a method in which only a subset of the genome is sequenced (typically 1%–10% [Lou et al. 2021]), rather than the entire genome (for some common examples see Albert et al. 2007; Choi et al. 2009; Peterson et al. 2012; Wallace and Mitchell 2017). This form of sequencing can be highly beneficial for population genetic studies because it allows for the recovery of a high number of SNPs while reducing sequencing cost. Moreover, compared to microsatellite analyses, this sequencing technique can allow researchers to sample fewer individuals as the increased number of variant sites allows for equal detection of population structure making it a highly effective technique when sample size is limited. Furthermore, reduced representation genome sequencing works well for large genomes or when there's no reference genome available. It can identify thousands of genetic variants while using less computing power than is needed for whole genome sequencing by focusing only on specific genome regions. In fact, even when a species has relatively high genetic diversity, thousands of SNP markers are better able to detect population structure and individual genetic diversity than microsatellite markers (e.g., Lemopoulos et al. 2019; Sunde et al. 2020). There are currently many methods available for generating these SNPs including: restriction associated DNA sequencing (RADseq) (Peterson et al. 2012), whole exome sequencing (Albert et al. 2007), exome capture (Choi et al. 2009) and genotype‐by‐sequencing (Wallace and Mitchell 2017). While this option often represents the best trade‐off between cost and the number of SNPs needed for fine‐scale population analysis, it is usually not suitable for selection studies because reduced representation genome sequencing is heavily biased toward neutral regions of the genome. *Whole genome sequencing* (WGS) is a form of DNA sequencing in which the entire genome is sequenced. This is often split into two types: (i) low coverage where the depth of coverage is (0.5 × −2×) (Lou et al. 2021) and (ii) high coverage where the depth of coverage is > 10×. The cost of low coverage whole genome sequencing is comparable to RADseq but covers the whole genome instead of 1–10% of the genome (Lou et al. 2021). Because of the breadth of sequencing, whole genome sequencing can be used for both adaptive and non‐adaptive population genetic studies. The depth of sequencing coverage means that new bioinformatic pipelines (e.g., ANGSD; Korneliussen et al. 2014) are used to identify genome‐wide SNPs based on a probabilistic framework and a reference genome, or a genome of a closely related species, is required. As sequencing costs continually decrease, the future may be moving to more frequent use of higher coverage whole genome sequencing.

Commonly, four main types of sequencing are used in studies of urban population genetics: (i) microsatellites, (ii) the sequencing of single genes (either nuclear or mitochondrial), (iii) reduced representation genome sequencing, and (iv) low coverage whole genome sequencing (Box 2). Microsatellites, when previously developed, are the most cost‐efficient (Guichoux et al. 2011; Antunes et al. 2022) while low coverage whole genome sequencing is significantly more expensive, with reduced representation genome sequencing varying depending on the method (Lou et al. 2021). When sample size is high, microsatellites are usually sufficient to detect fine scale population differences. However, as sample size decreases, a larger number of genetic markers are needed to detect population patterns and thus reduced representation genome sequencing or low coverage whole genome sequencing is a more efficient method. Finally, microsatellites require less computing power and bioinformatics knowledge, while greater computing power and bioinformatics knowledge are necessary when using reduced representation genome sequencing and low coverage whole genome sequencing.

Finally, it's important to recognize that numerous sequencing platforms exist and that these technologies are rapidly evolving, with frequent and substantial improvements. Sanger sequencing has been the sequencing platform for microsatellites and sequencing of single genes for decades, and is still a cost‐efficient option when sequencing is needed for very few loci or individuals. For larger datasets, researchers have used many “next generation sequencing” (NGS) platforms including Roche 454 (discontinued), IonTorrent (discontinued), Illumina (MySeq, HiSeq, NextSeq, etc.), PacBio, and Oxford Nanopore. Each of these platforms has pros and cons to sequencing, and we recommend consulting with Core Sequencing Facilities and/or sequencing vendors to identify which platform is the most cost‐efficient for the proposed study.

## 3 Studying Non‐Adaptive Variation in Urban Wildlife

Below we provide a common list of analysis techniques for exploring data. We intentionally do not explicitly state which analyses are best for a given set of data and instead recommend using several of these approaches to understand the story your data tells. We note that this is not a comprehensive list of all analyses, but instead a summary of common techniques used to study non‐adaptive genetic variation in wildlife.

### 3.1 Summary Statistics

When examining non‐adaptive variation in urban populations, one of the first steps is to calculate summary statistics. These statistics describe key information about the genetic diversity of the populations studied and can help determine how to run downstream analyses. Genetic diversity (defined in Box 3) is commonly measured as observed and/or expected heterozygosity (*H*_*o*_ & *H*_*e*_, respectively), allelic richness (*A*_*r*_ used for microsatellite data), percentage of polymorphic loci (, used for SNP data), and the inbreeding coefficient (*F*) (Freeland 2005). Sample sizes and genetic marker type will influence which summary statistics should be used. Genetic diversity measured from microsatellites will measure allelic richness (continuous variable) and heterozygosity per locus whereas SNP data will generally have far fewer alleles per locus (2–4, the number of possible nucleotides) with genetic diversity measured as theta pi () (e.g., Fischer et al. 2017). Comparing these summary statistics across sampled populations can provide researchers with insight into differences among populations.

BOX 3 **Definitions of common population genetic terminology.**

**Table:** 

*Admixture*—the genetic mixing of two (or more) previously isolated populations such that the resulting population is a blend of the source populations. *Allelic richness*—a measure of genetic variation across the population which represents the average number of alleles (variants) per genetic location (locus). *Bottleneck effect*—an evolutionary phenomenon in which a population's size is drastically reduced for one or more generations, leaving the resulting population with reduced genetic variation relative to the ancestral population. *Census population size (N*_*c*_ *)*—the total number of individuals in a population. *Effective population size (N*_*e*_ *)*—the number of individuals effectively contributing genes to the next generation. *Fixation index (F*_*ST*_ *)*—a measure of population differentiation. This measure is used in population structure analyses and is bounded from 0 to 1, with 0 as no structure (i.e., both sampled populations have equal allele frequencies) and 1 as highly structured (i.e., no shared alleles between two sampled populations). *Genetic diversity*—the total number of genetic variants (e.g., mutations) within a sample pool. *Genetic drift*—the stochastic change in frequency of an existing gene variant in a population. *Heterozygosity*—the condition in which an individual carries different alleles at a given locus (the opposite of homozygosity); observed heterozygosity is the proportion of individuals in the population that are heterozygous at a given locus. *Homozygosity*—the condition in which an individual carries a single allele at a given locus (the opposite of heterozygosity); observed homozygosity is the proportion of individuals in the population that are homozygosity at a given locus. *Inbreeding depression*—a reduction in the fitness of a population due to matings between closely related individuals leading to an increase in homozygosity and the expression of deleterious recessive alleles within a population. *Isolation by barrier*—Interruption of gene flow arising due to geographic or reproductive barriers. *Isolation by distance*—a pattern of increasing genetic dissimilarity positively correlated with geographic distance. *Isolation by resistance*—a model of isolation by distance which accounts for patterns of geographic heterogeneity impacting gene flow. *Linkage disequilibrium*—the nonrandom association of alleles at different loci that segregate together during meiosis. *Outbreeding depression*—a reduction in the fitness of a population due to the introduction of non‐adaptive variation and/or the disruption of locally adapted gene complexes. *Wahlund effect*—a reduction in observed heterozygosity relative to expectations due to the calculation of observed heterozygosity across a population composed of distinct subpopulations with different allele frequencies.

### 3.2 Population Size and Demographic History

Part of managing urban wildlife populations is determining the census population size (*N*_c_). One way to identify the *N*_c_ for cryptic species is by identifying the total number of individuals in a given region through genetic testing of scat (Mills et al. 2000; von Thaden et al. 2020). Another analysis to estimate the number of individuals in a population is through kinship analysis that can identify the number of breeding families within a population (Bravington et al. 2016). For example, Jasper et al. (2019) identified parent–offspring relationships of the yellow fever mosquito ( *Aedes aegypti* ) in urban high‐rise apartments in Malaysia and used these data to estimate the neighborhood size of 268 individuals within an area of 91 m, suggesting high densities of mosquitoes are found in relatively small geographic areas. While *N*_c_ can be important for tracking individuals, the effective population size (*N*_e_) more accurately estimates the number of individuals contributing to the next generation and, therefore, strongly influences the total genetic diversity. It is believed that a smaller effective population size may result in the accumulation of deleterious alleles, inbreeding depression, and reduced evolutionary potential due to lower genetic diversity (Charlesworth and Charlesworth 1987), which can be of particular importance for populations in decline. For example, the effective population size in the northern two‐lined salamander ( *Eurycea bislineata* ) was significantly smaller for salamanders caught in urban streams than those in suburban and rural streams around New York City, which poses a significant threat to the maintenance of genetic diversity in the small urban populations (Fusco et al. 2021). That said, inbreeding can also facilitate the purging of deleterious alleles from a population (Hedrick and Garcia‐Dorado 2016). As a result, a population may exhibit a low effective population size (*N*_e_) but show limited or no evidence of inbreeding depression (e.g., urban pest insects such as bed bugs, *Cimex* spp.) (Fountain et al. 2015; Hedrick and Garcia‐Dorado 2016). There are many methods in which researchers can estimate effective population size (Wang 2005) and one commonly used software is *NeEstimator* (v2; Do et al. 2014) that not only includes a graphical user interface but can also handle datasets that include more than 50,000 SNP loci.

Estimates of current *N*_e_ are affected by historical changes in demography (Mazet et al. 2016). For example, urban populations of monarch butterflies (*Dabaus plexippus*) have low *N*_e_ but these small populations are not necessarily due to urbanization since rural populations have similarly low *N*_e_ and demographic analyses indicate that these populations started to decline during the Last Glacial Maximum (LGM) (Miles, Carlen, et al. 2025). In contrast, the common bed bug ( *Cimex lectularius* ) experienced a sharp decline in *N*_e_ during the LGM. However, following the rise of civilization and the emergence of the first urban centers, its *N*_e_ rebounded dramatically—eventually surpassing any estimates from the past 60,000 years. In comparison, the *N*_e_ of the bat‐associated lineage of *C. lectularius* has continued to decline since the LGM without recovery, suggesting that urban‐association has facilitated the recovery of the human‐associated lineage (Miles, Carlen, et al. 2025). There are many demographic models that can estimate deep time changes in *N*_e_, such as pairwise sequentially Markovian coalescent model (Mather et al. 2020), as well as software that estimates changes on a more recent evolutionary timescale (1000 years to present) such as *Stairwayplot2* and *GONE* (Liu and Fu 2020; Santiago et al. 2020). One caveat to these recent demographic estimators is that generation time must be known (Mather et al. 2020; Liu and Fu 2020; Santiago et al. 2020) and they must be shorter than the timescale of urbanization to be able to detect a signal of urban influences on changes to the *N*_e_.

### 3.3 Population Structure and Connectivity

Part of urban wildlife management includes identifying populations and their relationships to each other. Researchers can choose to identify a population based on sampling locale or use programs that can identify populations based on shared genetic variation. When populations are identified a priori based on sampling locale, there is potential for introducing the Wahlund Effect, which is a reduction of heterozygosity or an excess of homozygotes due to population subdivision with the sampled “population” (see Zhivotovsky 2015). A variety of methods and programs can be used to identify population structure.

Classically, the fixation index (*F*_ST_) measures how different a priori defined populations are from each other and based on the breadth of molecular marker types and sample sizes, has had many iterations of equations to estimate this summary statistic (Wright 1943, 1965; Cockerham 1973; Weir and Cockerham 1984; Nei 1987; Excoffier et al. 1992). *F*_ST_ ranges from 0 to 1, where values closer to 0 suggest that populations have no structure and values closer to one mean that populations are genetically distinct from each other; however, even seemingly small values can be indicative of meaningful genetic differentiation among samples. For example, Miles et al. (2018) found that *F*_ST_ derived from Western black widow spider ( *Latrodectus hesperus* ) populations was lower between Phoenix, USA populations and Las Vegas, USA populations than either from Albuquerque populations. Booth et al. (2009) found that urban centers can act as significant barriers to gene flow in habitat generalist small mammals, resulting in higher *F*_ST_ values between populations separated by a city than between those that are geographically farther apart but separated by rural landscapes. *F*_ST_ also indicated that populations of pest species ubiquitous in urban centers are not necessarily exchanging genes freely, often exhibiting highly elevated values of *F*_ST_ (Saenz et al. 2012; Booth et al. 2012, 2016). There are many resources available to estimate *F*_ST_, including using *GenAlEx*, an Excel add‐on optimized for microsatellite data (Smouse et al. 2017), and packages in R such as *hierfstat* that can take both SNP and microsatellite data (Goudet 2005).

In urban contexts, exploring the genetic clustering of individuals and populations is a common research goal. The clustering of individuals in a principal component analysis (PCA) can help visualize population assignment. Moreover, this method is parameter‐free, without a priori assumptions of population identity allowing this method to be comparable across study species while also eliminating researcher bias (Jombart 2008; Novembre and Stephens 2008; Jombart et al. 2009, 2010). There are many R packages available to run PCAs, including (but not limited to) *gstudio* (Dyer 2009), and *adegenet* (Jombart 2008) and *vegan* (Dixon 2003). One caveat to using PCA to identify population assignment is that admixed or panmictic populations will look similar to each other in the PCA plot, preventing the researcher from knowing what evolutionary processes are leading to the observed phenomenon. For example, slum dwelling rats in Salvador, Brazil do not cluster by the valley from which they were collected, nor is there any strong clustering within the PCA (Kajdacsi et al. 2013). However, additional analyses show that while there are three genetic clusters, there is significant admixture among clusters (Kajdacsi et al. 2013). It is therefore important to consider multiple forms of analysis when drawing conclusions about population assignment. This can be addressed using software which use genetic clustering algorithms to reconstruct genetic ancestry, like *ADMIXTURE* and *STRUCTURE* (Pritchard et al. 2000; Alexander et al. 2009). For example, Schmitz et al. (2024) identified considerable overlap in their PCA analysis of *Arabadopsis thaliana* SNPs from across Europe, but found that the populations, although somewhat admixed, still showed population structure/clustering in their STRUCTURE analysis. On the other hand, Yakub and Tiffin (2017) found that their PCA and clustering analysis both revealed the same level of population structure in *Lepidium virginicum* , where urban populations were more closely related to each other than their nearest non‐urban pair.

Both *ADMIXTURE* and *STRUCTURE* can analyze microsatellite and SNP data and produce highly similar model outputs that are fitted to models. *STRUCTURE* employs a Bayesian approach with the option to include a priori information, whereas *ADMIXTURE* uses a maximum‐likelihood approach. Moreover, *STRUCTURE* models population structure and assigns the proportional likelihood that an individual is in a specific genetic cluster, while *ADMIXTURE* estimates the ancestry proportion of individuals. *fastSTRUCTURE* is similar to *STRUCTURE* but uses a variational inference approach as opposed to a strictly Bayesian approach, allowing it to more quickly analyze the data (Raj et al. 2014). Since many plants can reproduce asexually, INSTRUCT is another cluster identification program that uses the same Bayesian approach as STRUCTURE, but without the assumption of outbreeding (Gao et al. 2007). Additionally, *adegenet* has a similar analysis—genotype composition plot—which displays a barplot illustrating each individual's probability of assignment to multiple genetic clusters (Jombart 2008).

As mentioned previously, the genetic marker chosen can have wildly different outcomes. It is therefore important to consider multiple forms of analyses to compare clustering results across markers. For example, Munshi‐South et al. (2016) used *ADMIXTURE* and *fastSTRUCTURE* to analyze SNP data from white‐footed mice collected at urban, suburban, and rural locations in the Northeastern, USA (including New York City) and found that the mice formed two distinct genetic clusters. This was surprising given that previous work by Munshi‐South and Kharchenko (2010) had shown distinct genetic clustering by population of white‐footed mice across New York City, thus the researchers expected the more geographically expansive study in 2016 to identify even more genetic clusters. Munshi‐South et al. (2016) propose that the difference in results from these two studies is likely due to use of different genetic markers. Munshi‐South and Kharchenko (2010) used microsatellites which evolve faster than SNPs, and the use of SNPs in Munshi‐South et al. (2016) indicates that there is still ancestral variation (i.e., admixture) in white‐footed mouse populations.

### 3.4 Spatial Analysis

Before assessing the spatial genetic patterns of urban wildlife, it is essential to consider the geographic scale that is biologically relevant for the study organism. This is especially important for analyses that require direct input of landscape features. For example, an organism with a low dispersal ability will need a higher resolution landscape map than an organism with high dispersal ability. For example, Penone et al. (2013) found that the strength of the relationship between urbanization and variables such as species richness of Orthoptera became more negative with increasing scale, suggesting that at a fine scale the measure of species richness would have been significantly higher than that at a broader scale.

A common analysis when exploring spatial genetic structure of a population is to use a Mantel test to examine isolation by distance (IBD, Mantel 1967). A Mantel test evaluates the correlation between two distance matrices and is commonly used in urban population genetics to assess the relationship between genetic and geographic distances, including the strength and direction of these relationships. For example, Mueller et al. (2018) used a Mantel test to demonstrate that urban burrowing owls ( *Athene cunicularia* ) in Argentina showed signs of genetic isolation by distance over a relatively small geographic area, which includes two cities only 250 km apart. Similar to Mantel tests, Mantel correlograms can also be used to assess genetic isolation by distance; however, a Mantel correlogram divides the pairs of locations into distance classes and then performs a Mantel test on each distance class. This can provide more detail of how the relationship between geographic and genetic distances changes across spatial scales. For instance, Homola et al. (2019) used Mantel correlograms, which quantify relationships across various distance classes, to show that isolation by distance relationships were strongest at shorter distance classes for urban wood frogs ( *Lithobates sylvaticus* ) and spotted salamanders ( *Ambystoma maculatum* ) in Maine, USA. Such analysis may also assess resistance scenarios, where dispersal potential and rate may differ by landscape type (e.g., canopy cover) (McRae 2006). Both Mantel tests (Sokal and Rohlf 1995) and Mantel correlograms (Oden and Sokal 1986; Sokal 1986) can be run in the R packages *vegan* (Dixon 2003), *ecodist* (Goslee and Urban 2007) or *ade4* (Dray and Dufour 2007; Bougeard and Dray 2018; Chessel et al. 2004; Dray et al. 2007; Thioulouse et al. 2018), making them easily accessible to researchers conducting a first pass to understand the spatial genetic relationship in their data.

When investigating fine‐scale spatial genetic patterns, approaches that minimize variation within a cluster and maximize variation between clusters can be useful. Jombart (2008) developed the R package *adegenet* which, among many other functions, allows users to run a discriminant analysis of principal components (DAPC) and spatial analysis of principal components (sPCA). Discriminant analysis of principal components first transforms the data using a principal component analysis before performing a discriminant analysis to ensure that the variables submitted to the discriminant analysis are uncorrelated. Although not explicitly a spatial analysis, DAPC requires users to assign genetic clusters a priori, which are typically based on the geographic origin of the sampled individuals. For example, DeCandia et al. (2019) used DAPC to investigate patterns of urban colonization by red foxes ( *Vulpes vulpes* ) in Zurich, Switzerland, and identified five distinct genetic clusters. Richardson et al. (2017) used sPCA to detect a sharp genetic break in urban populations of brown rats living in three different valleys of the Pau da Lima neighborhood, Salvador, Brazil. Jombart (2012) and Jombart and Collins (2015) have developed excellent tutorials to help run these analyses on both microsatellite and SNP data; however, large SNP datasets (e.g., > > 10,000 SNPs) may need to be reduced to allow this package to run.

Another option for detecting fine‐scale spatial genetic patterns is to use a multivariate regression and Moran's Eigenvector Maps (MEM, Dray et al. 2006), like that employed in the R package *MEMGENE* (Galpern et al. 2014). *MEMGENE* will detect significant spatial patterns in genetic variation (estimated from either SNPs or microsatellites) that are statistically independent from each other and output these as *MEMGENE* variables that can then be visualized. For example, Combs et al. (2018) utilized *MEMGENE* to recover fine scale differences in brown rat spatial structure associated with roadways in Salvador, Brazil and Vancouver, Canada. The *MEMGENE* package also comes with an excellent tutorial to help users who are less familiar with analyzing these types of data (Galpern and Peres‐Neto 2014).

Fine‐scale spatial genetic patterns can be influenced by the spatial heterogeneity between populations. *Circuitscape* can be a valuable tool that uses microsatellites or SNP data as input to test hypotheses of isolation by resistance (McRae and Beier 2007). *Circuitscape* uses electrical circuit theory to model gene flow across the landscape by converting the landscape into high resistance landscape features and low resistance landscape features. For example, an urban stream salamander is expected to have difficulty crossing a paved road, therefore this landscape feature would get a high resistance value. A low resistance value would be assigned to streambeds, which are expected to be easy for a stream salamander to move through. With those caveats in mind, *Circuitscape* can still be a powerful tool to identify costs associated with moving across the urban landscape. Additionally, for *Circuitscape* results of genetic connectivity to be useful, users must have large computing power (at minimum, Linux/Ubuntu server with Intel 4144 CPU, 2.20GHz Clock speed, 20 cores, and 384 GB of RAM; www.github.com/Circuitescape) and highly accurate knowledge of how an organism interacts with different landscape features to inform parameter value input. For example, Braaker et al. (2017) used GPS tracking information from 40 European hedgehogs ( *Erinaceus europaeus* ) to assign resistance values to landscape features (e.g., lawn, footpath, forest, marsh, highway, etc.) in Zurich, Switzerland. They then used these resistance values to test the genetic connectivity of 147 hedgehogs, fitting models that allowed for multiple pathways that outperformed least‐cost pathways. However, the authors note that even given their robust datasets, GPS tracking of daily hedgehog movement and measures of gene flow, were best predicted by two different models (Braaker et al. 2017). *ResistanceGA*, an R package, is another valuable tool for assessing how genes move through the environment (Peterman 2018). This technique incorporates *Circuitscape* and least‐cost‐path models to estimate connectivity between samples; however, similar to *Circuitscape*, high computing power is needed and the choice of which environmental layers to include influences the results and can lead to incorrect assumptions by the model (Winiarski et al. 2020). Moreover, Daniel et al. (2025) suggested that sampling design impacts the optimization of isolation by resistance models, positing that further validation of true resistance values are required.

If there is little or no knowledge of landscape resistance, *Estimated Effective Migration Surfaces* (EEMS) or *Fast Estimated Effective Migration Surfaces* (FEEMS) can be useful for visualizing spatial patterns of genetic variation (Petkova et al. 2016; Marcus et al. 2021). Both programs use spatial models that allow migration rates to vary across the geography and produce heat maps showing areas where gene flow is higher than expected and lower than expected, with isolation by distance as the null model. Unlike *Circuitscape*, these programs can identify geographic barriers to gene flow that may not be obvious based solely on landscape features. While these programs are extremely similar, EEMS requires more computational time and processing power. *Fast Estimated Effective Migration Surfaces* is slightly less accurate but reduces computational complexity by avoiding full‐scale modeling, thereby decreasing computational time and processing power (Marcus et al. 2021). Although both programs can be used to assess spatial patterns using microsatellites or SNPs, knowledge of computer programming languages (e.g., Python, C++, Unix/Linux) is necessary to install and run EEMS or FEEMS.

### 3.5 Common Pitfalls When Studying Non‐Adaptive Genetic Variation

It is important for researchers to remember that population genetic analysis tools are models and may not reflect the true history of the population. All data analysis programs have assumptions, and understanding the biological relevance of the resulting model is essential. For example, genetic clustering programs such as *STRUCTURE* and *ADMIXTURE* are highly sensitive to isolation by distance and may find multiple genetic clusters when only a single, non‐panmictic genetic population exists across a large area (Meirmans 2012). Using a Mantel test when first exploring the data is one way to test explicitly for isolation by distance, and can be highly informative when considering the importance of a particular finding and informing hierarchical analyses. *STRUCTURE* analysis is also influenced by the presence of closely related individuals, resulting in inflated *K*‐values (where *K* is the number of genetic clusters explained by the data); each potentially representing familial clusters (Lawson et al. 2018). Similarly, DAPC is susceptible to a priori assumptions of populations because it specifically minimizes variation among individuals of the same group while maximizing variation between groups (Miller et al. 2020). Running multiple types of analysis is essential to gain a better understanding of the population genetic structure while considering the biology of an organism as this allows us to interpret results within our broader understanding of the natural world.

### 3.6 Simulated Example of Non‐Adaptive Variation

**FIGURE 2:**
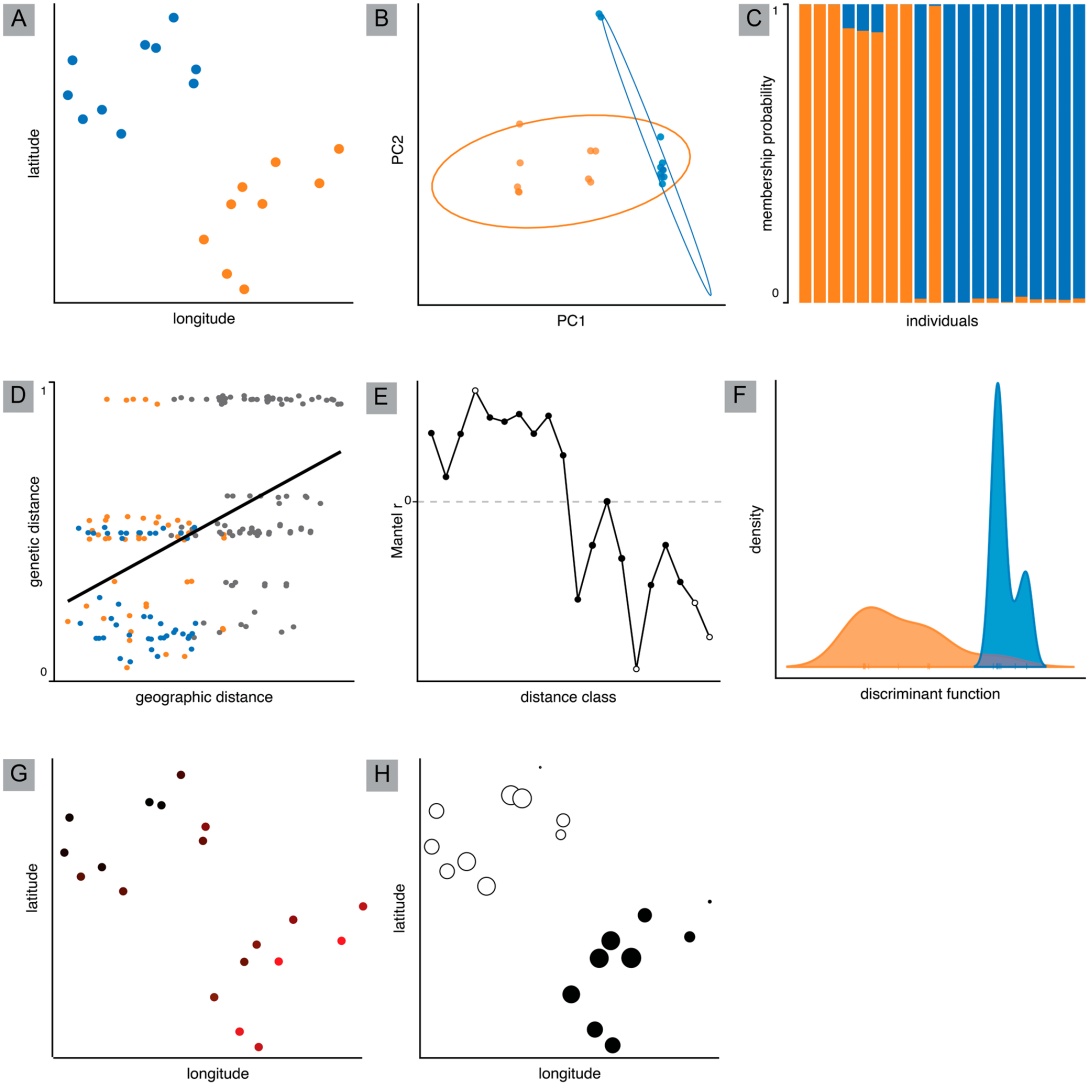
Example of non‐adaptive genetic variation data across two sampled populations. Here, we have simulated two populations that have diverged with equal rates of migration between them and 1000 variant sites (SNPs). (A) Simulated geographic location of each individual colored by population. (B) Principal component analysis with samples colored by location and ellipses representing 95% confidence of group assignment. (C) Genotype composition plot demonstrating how likely individuals are to be assigned to each colored population. (D) Visual representation of a Mantel test with blue and orange points representing within population comparisons, and gray points representing between population comparisons (i.e., between the blue and orange populations). The regression line shows the trend of increased geographic distance leading to increased genetic distance. (E) Mantel correlogram describing spatial autocorrelation, specifically showing correlations changes over geographic distance classes. Black points are statistically significant (*p* < 0.05), white points are non‐significant. (F) Discriminant analysis of principal components (DAPC) demonstrating clustering and separation of populations along the discriminant functions colored by sampling location. (G) Spatial principal component analysis with color representing the individual's sPCA score on the PC1, PC2, and PC3—colored red, green, and blue, respectively. (H) Moran's eigenvector maps demonstrating group assignment by color. Small white dots and small black dots are the most genetically similar, and large black dots and large white dots are the most genetically dissimilar.

To demonstrate the analyses of non‐adaptive variation described above, we simulated 1000 SNPs for 20 individuals across two populations (10 individuals per population) using the Hudson (2002) ms program. Additionally, we simulated geographic data and assigned each individual a location based on their population (Figure 2A). We then conducted a PCA (Figure 2B), genotype composition plot (Figure 2C), Mantel test (Figure 2D), Mantel correlogram (Figure 2E), DAPC (Figure 2F), sPCA (Figure 2G), and Moran's eigenvector maps (Figure 2H) to display the visualizations produced by each of these analyses. The code for the simulation and analyses can be found in supplement material.

## 4 Studying Adaptive Genetic Variation in Urban Wildlife

Similar to the above section on non‐adaptive evolution, below we provide a common list of analysis techniques for exploring data. Here we note that this is not a comprehensive list of all possible analyses, and in fact is rather scarce due to the limitations of identifying adaptive genomic variation in non‐model urban organisms. We emphasize that studies of genetic signatures of adaptations require either gene regions that have been previously well documented or well‐annotated reference genomes.

### 4.1 Role of Adaptive Variation in Wildlife

As we aim to sustain urban organisms, uncovering the genetic architecture of the urban phenotypes will allow us to better manage urban organisms (Winchell et al. 2022). In particular, it is important to determine what parts of the genome are under selection, the direction of the selection, and how quickly this section is occurring (Hendry et al. 2008). However, much of the current research on the genetic basis of adaptation has focused on model organisms, with few urban species having fully annotated genome sequences available. High throughput genome sequencing has reduced sequencing costs and allowed researchers working with non‐model organisms access to data once only available to well‐funded laboratories. Additionally, recent reviews have identified genes that may be under selection in urban organisms, allowing researchers with smaller budgets to tackle questions regarding adaptation by sequencing only the genes referenced instead of whole genomes (see Kreling et al. 2025 and Mueller et al. 2013 as examples). By leveraging these advances in genome sequencing and identifying key genes under selection, researchers are now better equipped to investigate urban adaptation in wildlife.

### 4.2 Identifying Adaptive Genetic Variation

Adaptive genetic variation can come in many forms including gene duplications (mutations that result in the copying of a segment of DNA), indels (genetic mutation involving the insertion or deletion of one or more nucleotides in a DNA sequence), and SNPs. One long standing method of identifying adaptive variation is to find SNPs in functional regions of known genes, also known as a candidate gene approach. For example, in the indoor urban pest the common bed bug ( *Cimex lectularius* ), there are three known SNP variants that confer resistance to insecticides that target the voltage gated sodium channel gene that are found at varying frequencies in the USA and Europe (Balvín and Booth 2018; Lewis et al. 2023; Booth 2024). Insecticides frequently used in urban areas will have a strong selective pressure on insect pests and have been developed to target specific gene pathways (Zhu et al. 2016). Because of this target pathway approach, researchers have been able to identify SNPs in these known genes that are adaptive in dealing with pesticide application (ffrench‐Constant et al. 2004). However, this adaptive SNP identification is not always feasible with non‐model organisms or non‐pests. Specifically, a known gene with a known variant must be previously sequenced or an annotated reference genome must be available. Adaptive genetic variation can only be identified in known gene coding regions of the genome.

Researchers can perform an *F*_ST_ outlier test between urban and non‐urban sampled populations on genome‐wide SNPs which identifies *F*_ST_ values that are either higher or lower than expected (Beaumont and Nichols 1997). If an annotated genome—or one from a closely related species—is available, researchers can examine the region containing the outlier SNP to determine whether it falls within a genic region. When performing an outlier *F*_ST_ test on reduced representation genome sequence, by virtue of being reduced representation, the likelihood of finding relevant genes is low (Rellstab et al. 2015; Lowry et al. 2017). However, when whole‐genome sequences are available alongside a well‐annotated reference genome (i.e., coding regions identified and gene products identified), researchers studying urban wildlife are more likely to detect genomic signatures of urban adaptation than using reduced representation or single gene sequencing approaches. For example, using whole genome sequences of brown rats in New York City researchers found multiple genes associated with rodenticide resistance under strong selection (Harpak et al. 2021). There are several programs available for performing *F*_ST_ outlier tests including R packages and command line programs (reviewed in Narum and Hess 2011).

Genome‐wide association studies (GWAS) identify loci under selection based on linkage disequilibrium that underlie phenotypic or environmental variation (guide available through https://github.com/MareesAT/GWA_tutorial/). These studies typically require sequencing the full genome, or many highly variable microsatellite markers, of many individuals (> 50 per phenotype/environment), with an annotated genome available (e.g., plink2; Chang et al. 2015). Additionally, some computational knowledge is needed as many of the programs are GNU/Linux‐based and some of the statistics are performed in R. Fisher's exact test is then used to test if there is enrichment for specific gene regions (Gaudet et al. 2017). For programs such as PLINK 2, it is recommended that populations are divergent, using top principal components as covariates, with highly related individuals removed, and the rare variants removed (Chang et al. 2015). Additional programs such as SNPTEST (Marchini et al. 2007) and GenABEL (Aulchenko et al. 2007) also analyze genome‐wide SNPs in a GWAS approach, but have yet to be implemented in urban adaptation studies. In GWAS studies, the functions of well‐annotated reference genes are known, thus researchers can then categorize these gene functions into groups and infer which urban factors (e.g., urban heat island, pollution, etc.) are influencing positive and purifying selection on these genes (Gaudet et al. 2017). For example, Mueller et al. (2020) sequenced 213 burrowing owls ( *Athene cunicularia* ) across three urban–rural population pairs. While they did not find single genomic sites under selection, they did find that gene sets associated with neurons and synapses were significantly enriched; given that urbanization is recent in this area, these findings suggest that selection has been acting on standing variation across these populations (Mueller et al. 2020).

Selective sweeps, where a beneficial mutation increases in frequency and becomes fixed, causes a reduction in genetic diversity in the surrounding genomic location (Stephan 2019). Identifying these positive selective sweeps requires a haplotype‐resolved annotated reference genome, often with a recombination map, and whole genome sequencing of samples that can be haplotype phased. This approach, similar to GWAS, requires population sampling to be done in pairs rather than along a transect as they test the “ancestral” alleles (e.g., non‐urban) against the derived allele (e.g., urban). Then programs such as selscan (Szpiech and Hernandez 2014), which are computationally extensive and require knowledge of bash script, can be used to estimate haplotype statistics (e.g., XP‐nSL, EHH, iHH12; Szpiech and Hernandez 2014; Sabeti et al. 2002; Voight et al. 2006) that detect selective sweeps in urban vs. non‐urban populations. For example, Santangelo et al. (2025), identified positive selection, but incomplete selective sweeps, between urban and rural populations of white clover, *Trifolium repens* , in Toronto, Ontario, Canada. Studies like these can better inform the interplay between the strength of selection and gene flow as previous studies on a single gene of interest, cyanide production (HCN), showed strong divergence between urban and non‐urban populations in Toronto (Thompson et al. 2016; Santangelo et al. 2022), suggesting that urbanization may act as a very strong selective force for white clover.

### 4.3 Common Pitfalls When Studying Adaptive Genetic Variation

The use of large SNP datasets may provide insightful information about the genetic composition of populations; however, by the very nature of being a large dataset (e.g., millions of variant sites), many statistical analyses may be significant (e.g., *p* < 0.001) but not necessarily biologically relevant (Sham and Purcell 2014; Kaler and Purcell 2019). For example, it is not uncommon in GWAS studies and *F*_ST_ outlier studies to find that a statistically significant SNP is found in a coding region of an uncharacterized gene product, especially in the era of newly published reference genomes (Rocha et al. 2023). Additionally, both *F*_ST_ outliers and GWAS studies test for directional selection (e.g., strong purifying selection) but cannot detect weak purifying selection which may be at play in urban environments. Directional/purifying selection relies on the outlier tests mentioned above. Balancing selection, on the other hand, is a result of selection favoring heterozygotes (Freeland 2005). As such, it is difficult to discern in the genome if the heterozygosity is due to balancing selection, standing variation with no/weak selection, or recent bottlenecks (Freeland 2005). Only recently have methods been generated and become available that are able to detect balancing selection and thus new research should also incorporate these (reviewed in Bitarello et al. 2023; Soni and Jensen 2024).

### 4.4 Case Study of Adaptive Variation

**FIGURE 3:**
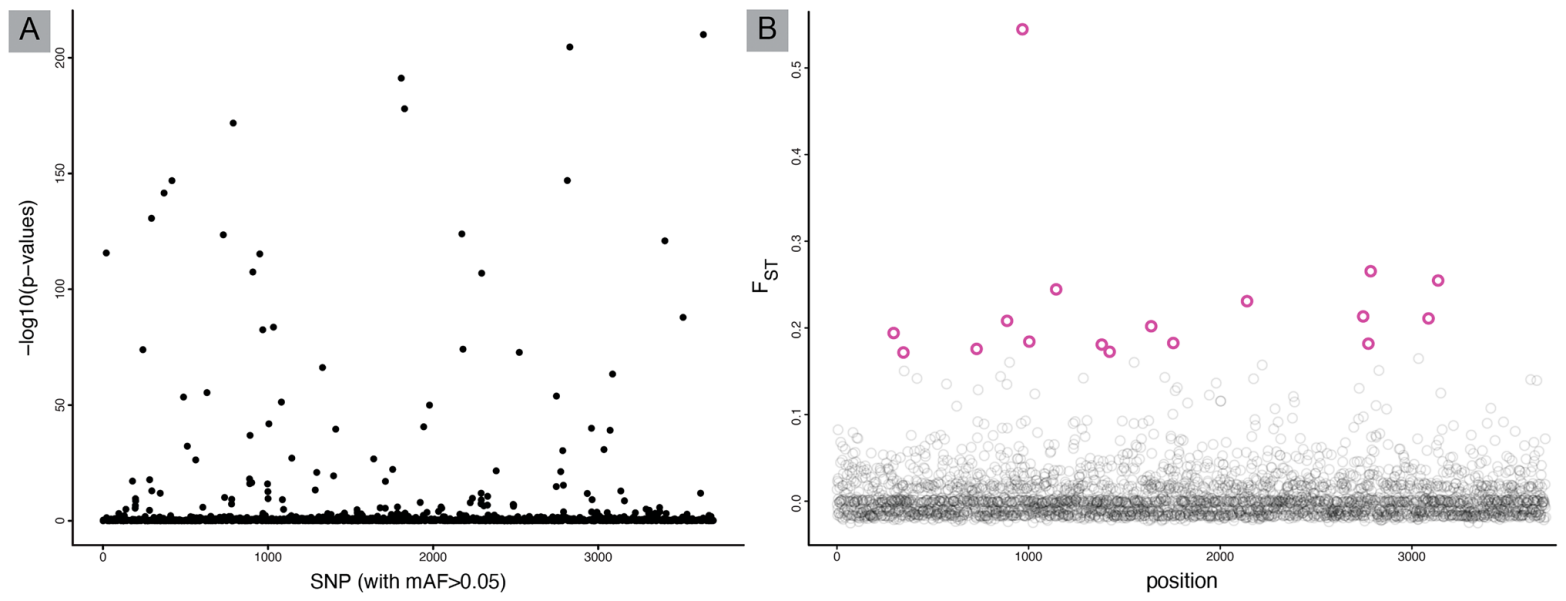
Example of adaptive genetic variation data across two sampled populations. Here, we use publicly available data (Xuereb et al. 2018) of Californian sea cucumber with 3699 SNPs. (A) SNP outlier detection which uses principal components to identify outliers among all sampled populations. In this image, all SNPs shown are outliers, and which SNPs are significant can be chosen by the researcher based on their preferred *p*‐value. (B) *F*_ST_ outlier detection using pairwise *F*_ST_ between two populations, this plot is similar to GWAS plots except *F*_ST_ outlier detection uses a priori population identification vs. GWAS uses phenotypic differences. Here, the purple circles represent the outlier SNPs that may be under selection. Researchers may benefit from using both analyses to determine which loci are consistently significant and thus most likely under selection.

To demonstrate the adaptive variation analyses described above, we used publicly available data from Xuereb et al. (2018) and the Marine Genomics 2021 online course material available on github (https://baylab.github.io/MarineGenomics/). The authors collected samples of Californian sea cucumber ( *Parastichopus californicus* ) from northern and southern regions of the northeastern Pacific Ocean and generated RADseq SNP data that yielded 3699 total SNPs. To identify potential adaptive loci, we conducted two types of SNP outlier detection analyses, one through PCA (Figure 3A) and one through *F*_ST_ (Figure 3B). The code for these analyses can be found in online supplement material.

## 5 Improving Connections Between Population Genomics and Informing Management Actions

While we recognize that there may be gaps in understanding how genetics applies to urban ecology, we appreciate the growing interest among urban ecologists in utilizing genomics tools. We believe that with further collaboration these tools can be applied effectively in the field allowing for conservation and preservation of urban organisms, while developing cities that benefit humans and nature.

### 5.1 Identifying Population Units

In managing populations, both pests and conservation, identifying population units is a key concern. As noted above, urban biologists can test whether their a priori defined populations are the same as a posteriori identified populations by examining patterns of population structure and connectivity using PCA and *STRUCTURE/ADMIXTURE* analyses. For example, streamside salamanders ( *Ambystoma barbouri* ) in urban areas across Tennessee, USA, form three clusters in both *STRUCTURE* and phylogenetic analyses, but were collected from 13 localities (Hubbs et al. 2022). Surprisingly, individuals from one of the localities clustered with individuals sampled further than individuals in a separate but geographically closer cluster. When identifying population units through genetic analyses, it is important to use multiple biologically relevant lines of evidence. Thus, population structure can be applied to identify management units, assess genetic diversity, and aid in management or recovery plans. In practice, these units can delineate the spatial scope of management and recovery plans, inform corridor and zoning overlays in comprehensive planning, and set the geographic basis for environmental review and mitigation.

### 5.2 Genetic Monitoring

Conventional monitoring techniques in urban wildlife can be particularly difficult when relying upon observation records, reports of road‐killed animals, and/or camera traps (which are prone to theft or destruction) (Meek et al. 2012). Recently, the use of invertebrate‐derived DNA (iDNA) and environmental DNA (eDNA) have become tools to detect biodiversity in urban areas (Kelly et al. 2016; Hoffmann et al. 2018). Some caveats to this method are that it relies on metabarcoding techniques, a technique in which the specific mitochondrial gene cytochrome oxidase 1 (CO1 also written as COX1 or COI) is amplified for animals. Metabarcoding can identify the potential presence of urban wildlife, but is limited to the availability of species specific metabarcodes and can be swamped by DNA from domestic animals (e.g., feral cats) living in urban areas (Hoffmann et al. 2018). Moreover, while this technique is useful for detecting the presence or absence of species, the DNA that is typically sequenced is highly conserved and therefore offer limited value for population‐level genetic analyses (Adams et al. 2019). To date, the use of eDNA to conduct population genetic studies has only been successful when significant resources have been invested in developing species‐specific sequencing techniques (Sigsgaard et al. 2016; Parsons et al. 2018).

Genetic monitoring also includes tracking levels of genetic diversity and identifying hybridization events within populations. As mentioned above, there are many ways to estimate genetic diversity from non‐invasively obtained (e.g., iDNA, eDNA) and less expensive genetic markers. If researchers take samples at multiple timepoints, they can track whether populations are gaining, maintaining, or losing genetic variation and adjust management strategies as needed. Additionally, if there is concern that hybridization events may be occurring, population genetic tools are available to assess this. For example, using a RADseq approach, Grabenstein et al. (2023) found that human disturbed habitats promoted hybridization between black‐capped ( *Poecile atricapillus* ) and mountain chickadees ( *Poecile gambeli* ). They identified hybrids through *STRUCTURE* analysis (noted above) and from calculating the hybrid index in the R package *gghybrid* (Grabenstein and Taylor 2018). As hybridization becomes increasingly common in urbanized and anthropogenically disturbed habitats (Grabenstein and Taylor 2018), researchers may benefit from the identification and management of these hybridization events. Hybrid indices can serve as compliance metrics in translocation permits and trigger containment or barrier modifications when thresholds are exceeded.

### 5.3 Managing Genetic Variants

Managing specific genetic variants can be part of urban conservation efforts as well as pest management. Identification of pesticide resistance‐associated mutations and population structure can aid in management decisions (Booth 2024). For example, many bed bug populations within the U.S. have been found to have mutations that confer resistance to pyrethroid insecticides (Lewis et al. 2023), however, within these same samples populations, mutations that confer resistance to phenylpyrazoles and cyclodienes are less common (Block et al. 2025); thus, genetic screening of problematic populations may assist managers in finding alternative insecticides that more effectively control infestations. Municipal integrated pest management (IPM) policies can codify rotation schemes based on local allele frequencies and set procurement standards that avoid chemistries with high resistance prevalence. Additionally population structure in pests, may elucidate cryptic movement between populations that managers might otherwise be unaware of (Booth et al. 2012; Combs et al. 2019; Fan et al. 2022). In conservation efforts, managers may want to eliminate maladaptive genetic variants that may cause populations to crash or find adaptive variants that allow them to persist (Stockwell et al. 2003; Derry et al. 2019). For example, researchers characterized the genome of the maritime pine ( *Pinus pinaster* ) and found the alleles correlated with survival in hot, arid regions (Jaramillo‐Correa et al. 2015). Jaramillo‐Correa et al. (2015) then used these candidate SNPs to predict forest density under different climate change scenarios. This allowed forest managers to decide if they wanted to increase local generic variation by introducing individuals from other climates or remove individuals that lack the desired genes for future climate predictions (Derry et al. 2019).

### 5.4 Assisted Gene Flow

Many urban populations suffer from intense habitat fragmentation, limiting their dispersal ability and potentially leading to genetic drift and inbreeding depression. One way to counteract this is via assisted gene flow, also known as genetic rescue, in which individuals from one geographic population are translocated to a different geographic population. For example, after studying the population genetics of the endangered San Francisco garter snake ( *Thamnophis sirtalis tetrataenia* ) found in two of California's most densely populated counties, Wood et al. (2020) recommended that one southern population could benefit from translocating individuals from a northern population. Importantly, Wood et al. (2020) note that it is critical to continue to genetically monitor the population after translocation to ensure there is no outbreeding depression or loss of local adaptation as well as observe any fitness differences such as population growth. Meanwhile, de Groot et al. (2016) found that European pine martens ( *Martes martes* ), which show spatial genetic structuring across Europe, have low levels of genetic divergence in the Netherlands, indicating that dispersal is occurring across one or more major urban areas including highways. The differences in the outcomes of these two studies emphasize the necessity of population genetic studies before translocation events.

### 5.5 Ex Situ Management

When urban wildlife populations decline due to total loss of habitat or exposure to environmental stressors (e.g., hunting, toxins, heat island effects, artificial light at night) captive breeding programs may be the only option until the threat is remediated. These programs can be highly successful if genetic monitoring is included when both removing individuals from the wild and releasing captive‐bred individuals back into the wild (Willoughby et al. 2017). Program authorization should require post‐release genomic assessment, with action thresholds linked to adaptive program changes. For example, the use of dichlorodiphenyltrichloroethane (DDT), an insecticide that had worldwide use during the 1950s and 1960s, led to widespread decline of many raptors, including peregrine falcons ( *Falco peregrinus* ) due to its accumulation in the food chain, resulting in egg‐shell thinning and widespread reproductive failures for the raptors. In the Midwestern USA, peregrine falcons were considered extirpated by 1964. However, since 1972, following the ban on the use of DDT in the USA, the captive breeding and reintroduction of these birds has led to robust urban populations throughout this region, even exceeding pre‐DDT population levels. Genetic analyses of the relationship among 350 peregrine falcons sampled across nine Midwestern USA cities found both high mate and nest‐site fidelity, female‐biased natal dispersal, and relatively high gene diversity, suggesting that the urban populations are behaving in a similar fashion to the non‐urban populations (Caballero et al. 2016). Thus, urbanization does not appear to be negatively impacting the behavior and ecology of the species, despite its initial extirpation due to anthropogenic factors. This highlights the need to reassess urban populations after captive breeding or other *ex situ* management.

### 5.6 Museum Specimens as Historic Reference

There has been an increasing interest in using museum specimens to identify phenotypic and genetic changes that have occurred in organisms between the past and current urban environments (Schultz et al. 2021; Card et al. 2021; Winchell et al. 2022). Due to DNA degradation, the optimal approach is to sequence whole genomes at high depth using an annotated reference genome for alignment (Axelsson et al. 2008). However, with limited resources to generate sequence data, other options exist, including target sequence capture (e.g., Bi et al. 2019), mitochondrial DNA sequencing (e.g., Richmond et al. 2017), and low coverage whole genome sequencing. These various sequencing methods can identify historic vs. contemporary population structure and signatures of adaptive selection. These tools, however, are limited by the availability of museum specimens and the quality of preservation that allows for sequencing (Schultz et al. 2021). However, where baseline data can be generated, these could be used to set quantitative restoration and offset targets that can be re‐assessed periodically during planning updates.

### 5.7 Incorporating Human Society

We encourage those who are working on the biology of urban wildlife to strongly consider how humans have shaped cities in the past and will continue to shape them in the future (Carlen et al. 2025). Researchers and managers should have a deep understanding of the social injustices that have influenced green space distribution, park management, roadway/highway placement, and resource access, as these policies directly structure the population genetics of urban organisms. For example, Schmidt and Garroway (2022) found that genetic diversity and habitat connectivity are lower in majority‐minority neighborhoods in the United States. Importantly, genomic patterns observed in one city are often not directly applicable to the same species in another. Miles et al. (2018) observed lower genetic diversity of Western black widow spiders in Albuquerque, USA compared to Las Vegas, USA or Phoenix, USA, likely due to the higher degree of tourism in Las Vegas and Phoenix. Similarly, Combs et al. (2018) demonstrated that genetic clustering of brown rats varied with city design, including the influence of major waterways in New Orleans, USA and roadway networks in Salvador, Brazil. These studies highlight how unique social histories and urban forms structure wildlife connectivity, adaptation, and diversity. Accordingly, genomic priorities should be paired with equity‐centered planning, directing investment (e.g., corridor development or habitat restoration) to neighborhoods with the lowest connectivity and genomic diversity, aligning with biodiversity outcomes with broader environmental goals.

## 6 Conclusions and Future Prospects

We have mentioned a number of spatial modeling tools for urban wildlife biologists, but many of these tools currently have limitations with regards to the total number of SNPs that can be analyzed. In this new era of big data, there is a growing need for landscape genomics to disentangle historical vs. contemporary spatial and genomic interactions (Bradburd and Ralph 2019). The use of museum specimens and whole genome sequencing is an exciting new venue to look at genetic variation that may have been lost in recent demographic events (Raxworthy and Smith 2021). Compared to Sanger‐sequencing techniques, advances in next generation sequencing have generated more accurate, faster, and less expensive sequencing (Satam et al. 2023) that make these urban population genetic studies attainable to broader groups of researchers.

New methods for big data are constantly being developed and it is important to keep up with advances in DNA sequencing and bioinformatic technology. In fact, as more data are generated, additional specialized expertise is required to manage and interpret them. For example, whole genome sequencing requires large computing power and knowledge of command line language. The biological significance of data requires knowledge of the organism and the environment that it dwells in. By strengthening interdisciplinary collaborations, we can overcome the challenges of methodological complexity and foster innovative solutions for urban conservation and management.

## Author Contributions

**Elizabeth J. Carlen:** conceptualization (equal), writing – original draft (equal), writing – review and editing (equal). **Lindsay S. Miles:** conceptualization (equal), writing – original draft (equal), writing – review and editing (equal). **Kevin Aviles‐Rodriguez:** conceptualization (equal), writing – review and editing (equal). **Warren Booth:** writing – review and editing (equal).

## Funding

This work was supported by the Living Earth Collaborative and the Joseph R. and Mary W. Wilson Urban Entomology Endowment.

## Conflicts of Interest

The authors declare no conflicts of interest.

## Supporting information


**Data S1:** ece373372‐sup‐0001‐supinfo.docx.

## Data Availability

The code for the simulation and analyses can be found in Supporting Information.

## References

[ece373372-bib-0001] Adams, C. I. M. , M. Knapp , N. J. Gemmell , et al. 2019. “Beyond Biodiversity: Can Environmental DNA (eDNA) Cut It as a Population Genetics Tool?” Genes 10: 192. 10.3390/genes10030192. 30832286 PMC6470983

[ece373372-bib-0002] Albert, T. J. , M. N. Molla , D. M. Muzny , et al. 2007. “Direct Selection of Human Genomic Loci by Microarray Hybridization.” Nature Methods 4, no. 11: 903–905. 17934467 10.1038/nmeth1111

[ece373372-bib-0003] Alexander, D. H. , J. Novembre , and K. Lange . 2009. “Fast Model‐Based Estimation of Ancestry in Unrelated Individuals.” Genome Research 19: 1655–1664. 10.1101/gr.094052.109.vidual. 19648217 PMC2752134

[ece373372-bib-0004] Allendorf, F. W. 2025. “Fifty Years of Conservation Genetics: A Personal Perspective.” Molecular Ecology 34: e17705. 10.1111/mec.17705. 40026218

[ece373372-bib-0005] Andrés, J. A. , and S. M. Bogdanowicz . 2011. “Isolating Microsatellite Loci: Looking Back, Looking Ahead.” In Molecular Methods for Evolutionary Genetics, edited by V. Orgogozo and M. V. Rockman , 211–232. Humana Press. 10.1007/978-1-61779-228-1_12 22065440

[ece373372-bib-0006] Antunes, A. M. , J. G. Nunes Stival , C. P. Targueta , M. P. de Campos Telles , and T. N. Soares . 2022. “A Pipeline for the Development of Microsatellite Markers Using Next Generation Sequencing Data.” Current Genomics 23: 175–181. 10.2174/1389202923666220428101350. 36777003 PMC9878831

[ece373372-bib-0007] Aulchenko, Y. S. , S. Ripke , A. Isaacs , and C. M. Van Duijn . 2007. “GenABEL: An R Library for Genome‐Wide Association Analysis.” Bioinformatics 23, no. 10: 1294–1296. 17384015 10.1093/bioinformatics/btm108

[ece373372-bib-0008] Avise, J. C. 2012. Molecular Markers, Natural History and Evolution. Springer Science & Business Media.

[ece373372-bib-0183] Axelsson, E. , M. Eske Willerslev , T. P. Gilbert , and R. Nielsen . 2008. “The Effect of Ancient DNA Damage on Inferences of Demographic Histories.” Molecular Biology and Evolution 25, no. 10: 2181–2187. 18653730 10.1093/molbev/msn163

[ece373372-bib-0009] Balvín, O. , and W. Booth . 2018. “Distribution and Frequency of Pyrethroid Resistance‐Associated Mutations in Host Lineages of the Bed Bug (Hemiptera: Cimicidae) Across Europe.” Journal of Medical Entomology 55: 923–928. 10.1093/jme/tjy023. 29562293

[ece373372-bib-0010] Beaumont, M. A. , and R. A. Nichols . 1997. “Evaluating Loci for Use in the Genetic Analysis of Population Structure.” Proceedings of the Royal Society of London. Series B, Biological Sciences 263: 1619–1626. 10.1098/rspb.1996.0237.

[ece373372-bib-0011] Bi, K. , T. Linderoth , S. Singhal , et al. 2019. “Temporal Genomic Contrasts Reveal Rapid Evolutionary Responses in an Alpine Mammal During Recent Climate Change.” PLoS Genetics 15: e1008119. 10.1371/journal.pgen.1008119. 31050681 PMC6519841

[ece373372-bib-0012] Bitarello, B. D. , D. Y. C. Brandt , D. Meyer , and A. M. Andrés . 2023. “Inferring Balancing Selection From Genome‐Scale Data.” Genome Biology and Evolution 15: evad032. 10.1093/gbe/evad032. 36821771 PMC10063222

[ece373372-bib-0013] Block, C. J. , L. S. Miles , C. D. Lewis , C. Schal , E. L. Vargo , and W. Booth . 2025. “First Evidence of the A302S Rdl Insecticide Resistance Mutation in Populations of the Bed Bug, *Cimex lectularius* (Hemiptera: Cimicidae) in North America.” Journal of Medical Entomology 62: tjaf033. 10.1093/jme/tjaf033. 40084570

[ece373372-bib-0014] Booth, W. 2024. “Population Genetics as a Tool to Understand Invasion Dynamics and Insecticide Resistance in Indoor Urban Pest Insects.” Current Opinion in Insect Science 62: 101166. 10.1016/j.cois.2024.101166. 38253200

[ece373372-bib-0015] Booth, W. , W. I. Montgomery , and P. A. Prodöhl . 2009. “Spatial Genetic Structuring in a Vagile Species, the European Wood Mouse.” Journal of Zoology 279: 219–228. 10.1111/j.1469-7998.2009.00608.x.

[ece373372-bib-0016] Booth, W. , V. L. Saenz , R. G. Santangelo , C. Wang , C. Schal , and E. L. Vargo . 2012. “Molecular Markers Reveal Infestation Dynamics of the Bed Bug (Hemiptera: Cimicidae) Within Apartment Buildings.” Journal of Medical Entomology 49: 535–546. 10.1603/me11256. 22679860

[ece373372-bib-0017] Booth, W. , C. Schal , and E. L. Vargo . 2016. “Population Genetics of Bed Bugs.” In Advances in the Biology and Management of Modern Bed Bugs, edited by S. L. Doggett , D. M. Miller , and C.‐Y. Lee . John Wiley & Sons. Inc.

[ece373372-bib-0018] Bougeard, S. , and S. Dray . 2018. “Supervised Multiblock Analysis in R With the ade4 Package.” Journal of Statistical Software 86, no. 1: 1–17. 10.18637/jss.v086.i01.

[ece373372-bib-0019] Braaker, S. , U. Kormann , F. Bontadina , and M. K. Obrist . 2017. “Prediction of Genetic Connectivity in Urban Ecosystems by Combining Detailed Movement Data, Genetic Data and Multi‐Path Modelling.” Landscape and Urban Planning 160: 107–114. 10.1016/j.landurbplan.2016.12.011.

[ece373372-bib-0020] Bradburd, G. S. , and P. L. Ralph . 2019. “Spatial Population Genetics: It's About Time.” Annual Review of Ecology, Evolution, and Systematics 50: 427–449. 10.1146/annurev-ecolsys-110316-022659.

[ece373372-bib-0021] Bravington, M. V. , H. J. Skaug , and E. C. Anderson . 2016. “Close‐Kin Mark‐Recapture.” Statistical Science 31: 259–274. 10.1214/16-STS552.

[ece373372-bib-0022] Caballero, I. C. , J. M. Bates , M. Hennen , and M. V. Ashley . 2016. “Sex in the City: Breeding Behavior of Urban Peregrine Falcons in the Midwestern US.” PLoS One 11: e0159054. 10.1371/journal.pone.0159054. 27420915 PMC4946791

[ece373372-bib-0023] Card, D. C. , B. Shapiro , G. Giribet , C. Moritz , and S. V. Edwards . 2021. “Museum Genomics.” Annual Review of Genetics 55: 633–659. 10.1146/annurev-genet-071719-020506. 34555285

[ece373372-bib-0024] Carlen, E. J. , A. Caizergues , Z. Jagiello , et al. 2025. “Legacy Effects of Religion, Politics, and War on Urban Evolutionary Biology.” Nature Cities 2: 593–602. 10.1038/s44284-025-00249-3.

[ece373372-bib-0025] Carlen, E. , and J. Munshi‐South . 2021. “Widespread Genetic Connectivity of Feral Pigeons Across the Northeastern Megacity.” Evolutionary Applications 14: 150–162. 10.1111/eva.12972. 33519962 PMC7819573

[ece373372-bib-0026] Castoe, T. A. , A. W. Poole , A. P. J. de Koning , et al. 2012. “Rapid Microsatellite Identification From Illumina Paired‐End Genomic Sequencing in Two Birds and a Snake.” PLoS One 7: e30953. 10.1371/journal.pone.0030953. 22348032 PMC3279355

[ece373372-bib-0027] Chang, C. C. , C. C. Chow , L. C. C. A. M. Tellier , et al. 2015. “Second‐Generation PLINK: Rising to the Challenge of Larger and Richer Datasets.” GigaScience 4: 1–16. 10.1186/s13742-015-0047-8. 25722852 PMC4342193

[ece373372-bib-0028] Charlesworth, D. , and B. Charlesworth . 1987. “Inbreeding Depression and Its Evolutionary Consequences.” Annual Review of Ecology and Systematics 18: 237–268.

[ece373372-bib-0029] Chessel, D. , A. Dufour , and J. Thioulouse . 2004. “The ade4 Package – I: One‐Table Methods.” R News 4, no. 1: 5–10. https://cran.r‐project.org/doc/Rnews/.

[ece373372-bib-0030] Choi, M. , U. I. Scholl , W. Ji , et al. 2009. “Genetic Diagnosis by Whole Exome Capture and Massively Parallel DNA Sequencing.” Proceedings of the National Academy of Sciences 106, no. 45: 19096–19101. 10.1073/pnas.0910672106 PMC2768590 19861545

[ece373372-bib-0031] Cockerham, C. C. 1973. “Analysis of Gene Frequencies.” Genetics 74: 679–700. 10.1093/genetics/74.4.679. 17248636 PMC1212983

[ece373372-bib-0032] Collins, M. K. , S. B. Magle , and T. Gallo . 2021. “Global Trends in Urban Wildlife Ecology and Conservation.” Biological Conservation 261: 109236. 10.1016/j.biocon.2021.109236.

[ece373372-bib-0033] Combs, M. , K. Byers , C. Himsworth , and J. Munshi‐South . 2019. “Harnessing Population Genetics for Pest Management: Theory and Application for Urban Rats.” Human‐Wildlife Interactions 13, no. 2: 250–263.

[ece373372-bib-0034] Combs, M. , K. A. Byers , B. M. Ghersi , et al. 2018. “Urban Rat Races: Spatial Population Genomics of Brown Rats ( *Rattus norvegicus* ) Compared Across Multiple Cities.” Proceedings of the Royal Society of London. Series B, Biological Sciences 285: 20180245. 10.1098/rspb.2018.0245. PMC6015871 29875297

[ece373372-bib-0035] Crissman, J. R. , W. Booth , R. G. Santangelo , et al. 2010. “Population Genetic Structure of the German Cockroach (Blattodea: Blattellidae) in Apartment Buildings.” Journal of Medical Entomology 47: 553–564. 10.1603/ME09036. 20695270 PMC7027314

[ece373372-bib-0036] Daniel, A. , P. Savary , J.‐C. Foltête , et al. 2025. “What Can Optimized Cost Distances Based on Genetic Distances Offer? A Simulation Study on the Use and Misuse of ResistanceGA.” Molecular Ecology Resources 25: e14024. 10.1111/1755-0998.14024. 39417711 PMC11646299

[ece373372-bib-0037] de Groot, G. A. , T. R. Hofmeester , M. La Haye , et al. 2016. “Hidden Dispersal in an Urban World: Genetic Analysis Reveals Occasional Long‐Distance Dispersal and Limited Spatial Substructure Among Dutch Pine Martens.” Conservation Genetics 17: 111–123. 10.1007/s10592-015-0765-6.

[ece373372-bib-0038] DeCandia, A. L. , K. E. Brzeski , E. Heppenheimer , et al. 2019. “Urban Colonization Through Multiple Genetic Lenses: The City‐ Fox Phenomenon Revisited.” Ecology and Evolution 9: 2046–2060. 30847091 10.1002/ece3.4898 PMC6392345

[ece373372-bib-0039] Derry, A. M. , D. J. Fraser , S. P. Brady , et al. 2019. “Conservation Through the Lens of (Mal)adaptation: Concepts and Meta‐Analysis.” Evolutionary Applications 12: 1287–1304. 10.1111/eva.12791. 31417615 PMC6691223

[ece373372-bib-0040] Dixon, P. 2003. “VEGAN, a Package of R Functions for Community Ecology.” Journal of Vegetation Science 14, no. 6: 927–930.

[ece373372-bib-0041] Do, C. , R. S. Waples , D. Peel , et al. 2014. “NeEstimator v2: Re‐Implementation of Software for the Estimation of Contemporary Effective Population Size (Ne) From Genetic Data.” Molecular Ecology Resources 14: 209–214. 10.1111/1755-0998.12157. 23992227

[ece373372-bib-0042] Dray, S. , and A. Dufour . 2007. “The ade4 Package: Implementing the Duality Diagram for Ecologists.” Journal of Statistical Software 22, no. 4: 1–20. 10.18637/jss.v022.i04.

[ece373372-bib-0043] Dray, S. , A. Dufour , and D. Chessel . 2007. “The ade4 Package – II: Two‐Table and K‐Table Methods.” R News 7, no. 2: 47–52. https://cran.r‐project.org/doc/Rnews/.

[ece373372-bib-0044] Dray, S. , P. Legendre , and P. R. Peres‐Neto . 2006. “Spatial Modelling: A Comprehensive Framework for Principal Coordinate Analysis of Neighbour Matrices (PCNM).” Ecological Modelling 196, no. 3–4: 483–493.

[ece373372-bib-0045] Dyer, R. J. 2009. “GeneticStudio: A Suite of Programs for Spatial Analysis of Genetic‐Marker Data.” Molecular Ecology Resources 9, no. 1: 110–113. 21564574 10.1111/j.1755-0998.2008.02384.x

[ece373372-bib-0046] Excoffier, L. , P. E. Smouse , and J. M. Quattro . 1992. “Analysis of Molecular Variance Inferred From Metric Distances Among DNA Haplotypes: Application to Human Mitochondrial DNA Restriction Data.” Genetics 131: 479–491. 10.1093/genetics/131.2.479. 1644282 PMC1205020

[ece373372-bib-0047] Fan, X. , C. Wang , and D. E. Bunker . 2022. “Population Structure of German Cockroaches (Blattodea: Ectobiidae) in an Urban Environment Based on Single Nucleotide Polymorphisms.” Journal of Medical Entomology 59: 1319–1327. 35462399 10.1093/jme/tjac036

[ece373372-bib-0048] ffrench‐Constant, R. H. , P. J. Daborn , and G. L. Goff . 2004. “The Genetics and Genomics of Insecticide Resistance.” Trends in Genetics 20: 163–170. 10.1016/j.tig.2004.01.003. 15036810

[ece373372-bib-0049] Fischer, M. C. , C. Rellstab , M. Leuzinger , et al. 2017. “Estimating Genomic Diversity and Population Differentiation – An Empirical Comparison of Microsatellite and SNP Variation in Arabidopsis Halleri.” BMC Genomics 18: 69. 10.1186/s12864-016-3459-7. 28077077 PMC5225627

[ece373372-bib-0050] Flores‐Manzanero, A. , D. Valenzuela‐Galvan , A. D. Cuaron , and E. Vazquez‐Dominguez . 2022. “Conservation Genetics of Two Critically Endangered Island Dwarf Carnivores.” Conservation Genetics 23: 35–49. 10.1007/s10592-021-01401-x.

[ece373372-bib-0051] Fountain, T. , R. K. Butlin , K. Reinhardt , and O. Otti . 2015. “Outbreeding Effects in an Inbreeding Insect, *Cimex lectularius* .” Ecology and Evolution 5: 409–418. 10.1002/ece3.1373. 25691967 PMC4314272

[ece373372-bib-0052] Freeland, J. R. 2005. Molecular Ecology. John Wiley & Sons, Ltd.

[ece373372-bib-0053] Fusco, N. A. , E. Pehek , and J. Munshi‐South . 2021. “Urbanization Reduces Gene Flow but Not Genetic Diversity of Stream Salamander Populations in the New York City Metropolitan Area.” Evolutionary Applications 14, no. 1: 99–116. 33519959 10.1111/eva.13025 PMC7819553

[ece373372-bib-0054] Galpern, P. , and P. Peres‐Neto . 2014. “MEMGENE Package for R: Tutorials.”

[ece373372-bib-0055] Galpern, P. , P. R. Peres‐Neto , J. Polfus , and M. Manseau . 2014. “MEMGENE: Spatial Pattern Detection in Genetic Distance Data.” Methods in Ecology and Evolution 5: 1116–1120. 10.1111/2041-210X.12240.

[ece373372-bib-0056] Gao, H. , S. Williamson , and C. D. Bustamante . 2007. “A Markov Chain Monte Carlo Approach for Joint Inference of Population Structure and Inbreeding Rates From Multilocus Genotype Data.” Genetics 176, no. 3: 1635–1651. 17483417 10.1534/genetics.107.072371 PMC1931536

[ece373372-bib-0057] Gaudet, P. , N. Škunca , J. C. Hu , and C. Dessimoz . 2017. “Primer on the Gene Ontology.” In The Gene Ontology Handbook, 25–37. Springer New York. 10.1007/978-1-4939-3743-1_3 PMC6377150 27812933

[ece373372-bib-0058] Goslee, S. C. , and D. L. Urban . 2007. “The Ecodist Package for Dissimilarity‐Based Analysis of Ecological Data.” Journal of Statistical Software 22: 1–19. 10.18637/jss.v022.i07.

[ece373372-bib-0059] Goudet, J. 2005. “Hierfstat, a Package for r to Compute and Test Hierarchical F‐Statistics.” Molecular Ecology Notes 5: 184–186. 10.1111/j.1471-8286.2004.00828.x.

[ece373372-bib-0060] Grabenstein, K. C. , K. A. Otter , T. M. Burg , and S. A. Taylor . 2023. “Hybridization Between Closely Related Songbirds Is Related to Human Habitat Disturbance.” Global Change Biology 29: 955–968. 10.1111/gcb.16476. 36305309

[ece373372-bib-0061] Grabenstein, K. C. , and S. A. Taylor . 2018. “Breaking Barriers: Causes, Consequences, and Experimental Utility of Human‐Mediated Hybridization.” Trends in Ecology & Evolution 33: 198–212. 10.1016/j.tree.2017.12.008. 29306562

[ece373372-bib-0062] Grimm, N. B. , S. H. S. H. Faeth , N. E. Golubiewski , et al. 2008. “Global Change and the Ecology of Cities.” Science 319: 756–760. 10.1126/science.1150195. 18258902

[ece373372-bib-0063] Guichoux, E. , L. Lagache , S. Wagner , et al. 2011. “Current Trends in Microsatellite Genotyping.” Molecular Ecology Resources 11: 591–611. 10.1111/j.1755-0998.2011.03014.x. 21565126

[ece373372-bib-0064] Harpak, A. , N. Garud , N. A. Rosenberg , et al. 2021. “Genetic Adaptation in New York City Rats.” Genome Biology and Evolution 13: evaa247. 10.1093/gbe/evaa247. 33211096 PMC7851592

[ece373372-bib-0065] Hedrick, P. W. , and A. Garcia‐Dorado . 2016. “Understanding Inbreeding Depression, Purging, and Genetic Rescue.” Trends in Ecology & Evolution 31: 940–952. 10.1016/j.tree.2016.09.005. 27743611

[ece373372-bib-0066] Hendry, A. P. , T. J. Farrugia , and M. T. Kinnison . 2008. “Human Influences on Rates of Phenotypic Change in Wild Animal Populations.” Molecular Ecology 17, no. 1: 20–29. 18173498 10.1111/j.1365-294X.2007.03428.x

[ece373372-bib-0067] Hoffmann, C. , K. Merkel , A. Sachse , P. Rodríguez , F. H. Leendertz , and S. Calvignac‐Spencer . 2018. “Blow Flies as Urban Wildlife Sensors.” Molecular Ecology Resources 18: 502–510. 10.1111/1755-0998.12754. 29328547

[ece373372-bib-0068] Hohenlohe, P. A. , W. C. Funk , and O. P. Rajora . 2021. “Population Genomics for Wildlife Conservation and Management.” Molecular Ecology 30: 62–82. 10.1111/mec.15720. 33145846 PMC7894518

[ece373372-bib-0069] Homola, J. J. , C. S. Loftin , and M. T. Kinnison . 2019. “Landscape Genetics Reveals Unique and Shared Effects of Urbanization for Two Sympatric Pool‐Breeding Amphibians.” Ecology and Evolution 9: 11799–11823. 10.1002/ece3.5685. 31695889 PMC6822048

[ece373372-bib-0070] Hubbs, N. W. , C. R. Hurt , J. Niedzwiecki , B. Leckie , and D. Withers . 2022. “Conservation Genomics of Urban Populations of Streamside Salamander ( *Ambystoma barbouri* ).” PLoS One 17: e0260178. 10.1371/journal.pone.0260178. 35771804 PMC9246143

[ece373372-bib-0071] Hudson, R. R. 2002. “Generating Samples Under a Wright‐Fisher Neutral Model.” Bioinformatics 18: 337–338. 11847089 10.1093/bioinformatics/18.2.337

[ece373372-bib-0072] IUCN SSC Amphibian Specialist Group . 2023. “ *Salamandra salamandra* .” The IUCN Red List of Threatened Species 2023: e.T59467A219148292. 10.2305/IUCN.UK.2023-1.RLTS.T59467A219148292.en.

[ece373372-bib-0073] Jaramillo‐Correa, J. P. , I. Rodríguez‐Quilón , D. Grivet , et al. 2015. “Molecular Proxies for Climate Maladaptation in a Long‐Lived Tree ( *Pinus pinaster* Aiton, Pinaceae).” Genetics 199, no. 3: 793–807. 10.1534/genetics.114.173252. 25549630 PMC4349072

[ece373372-bib-0074] Jasper, M. , T. L. Schmidt , N. W. Ahmad , S. P. Sinkins , and A. A. Hoffmann . 2019. “A Genomic Approach to Inferring Kinship Reveals Limited Intergenerational Dispersal in the Yellow Fever Mosquito.” Molecular Ecology Resources 19: 1254–1264. 10.1111/1755-0998.13043. 31125998 PMC6790672

[ece373372-bib-0075] Johnson, M. T. J. , and J. Munshi‐South . 2017. “Evolution of Life in Urban Environments.” Science 358: eaam8327. 10.1126/science.aam8327. 29097520

[ece373372-bib-0076] Jombart, T. 2008. “Adegenet: A R Package for the Multivariate Analysis of Genetic Markers.” Bioinformatics 24: 1403–1405. 10.1093/bioinformatics/btn129. 18397895

[ece373372-bib-0077] Jombart, T. 2012. “A Tutorial for the Spatial Analysis of Principal Components (sPCA) Using Adegenet 1.3‐6.”

[ece373372-bib-0078] Jombart, T. , and C. Collins . 2015. “A Tutorial for Discriminant Analysis of Principal Components (DAPC) Using Adegenet 2.1.6.”

[ece373372-bib-0079] Jombart, T. , S. Devillard , and F. Balloux . 2010. “Discriminant Analysis of Principal Components: A New Method for the Analysis of Genetically Structured Populations.” BMC Genetics 11: 206–209. 10.1109/MWP.2013.6724056. PMC2973851 20950446

[ece373372-bib-0080] Jombart, T. , D. Pontier , and A.‐B. Dufour . 2009. “Genetic Markers in the Playground of Multivariate Analysis.” Heredity 102: 330–341. 10.1038/hdy.2008.130. 19156164

[ece373372-bib-0081] Kajdacsi, B. , F. Costa , C. Hyseni , et al. 2013. “Urban Population Genetics of Slum‐Dwelling Rats ( *Rattus norvegicus* ) in Salvador, Brazil.” Molecular Ecology 22: 5056–5070. 10.1111/mec.12455. 24118116 PMC3864905

[ece373372-bib-0082] Kaler, A. S. , and L. C. Purcell . 2019. “Estimation of a Significance Threshold for Genome‐Wide Association Studies.” BMC Genomics 20: 618. 10.1186/s12864-019-5992-7. 31357925 PMC6664749

[ece373372-bib-0083] Kelly, R. P. , J. L. O'Donnell , N. C. Lowell , et al. 2016. “Genetic Signatures of Ecological Diversity Along an Urbanization Gradient.” PeerJ 4: e2444. 10.7717/peerj.2444. 27672503 PMC5028742

[ece373372-bib-0084] Korneliussen, T. S. , A. Albrechtsen , and R. Nielsen . 2014. “ANGSD: Analysis of Next Generation Sequencing Data.” BMC Bioinformatics 15: 356. 10.1186/s12859-014-0356-4. 25420514 PMC4248462

[ece373372-bib-0085] Kreling, S. E. S. , S. E. Vance , and E. J. Carlen . 2025. “Adaptation in the Alleyways: Candidate Genes Under Potential Selection in Urban Coyotes.” Genome Biology and Evolution 17: evae279. 10.1093/gbe/evae279. 39786569 PMC11775663

[ece373372-bib-0086] Lawson, D. J. , L. van Dorp , and D. Falush . 2018. “A Tutorial on How Not to Over‐Interpret STRUCTURE and ADMIXTURE Bar Plots.” Nature Communications 9: 1–11. 10.1038/s41467-018-05257-7. PMC6092366 30108219

[ece373372-bib-0087] Lemopoulos, A. , J. M. Prokkola , S. Uusi‐Heikkilä , et al. 2019. “Comparing RADseq and Microsatellites for Estimating Genetic Diversity and Relatedness — Implications for Brown Trout Conservation.” Ecology and Evolution 9: 2106–2120. 10.1002/ece3.4905. 30847096 PMC6392366

[ece373372-bib-0088] Lewis, C. D. , B. A. Levine , C. Schal , E. L. Vargo , and W. Booth . 2023. “Decade Long Upsurge in Mutations Associated With Pyrethroid Resistance in Bed Bug Populations in the USA.” Journal of Pest Science 96: 415–423. 10.1007/s10340-022-01505-4.

[ece373372-bib-0089] Liu, X. , and Y.‐X. Fu . 2020. “Stairway Plot 2: Demographic History Inference With Folded SNP Frequency Spectra.” Genome Biology 21: 280. 10.1186/s13059-020-02196-9. 33203475 PMC7670622

[ece373372-bib-0090] Lou, R. N. , A. Jacobs , A. P. Wilder , and N. O. Therkildsen . 2021. “A Beginner's Guide to Low‐Coverage Whole Genome Sequencing for Population Genomics.” Molecular Ecology 30: 5966–5993. 10.1111/mec.16077. 34250668

[ece373372-bib-0091] Lourenço, A. , D. Álvarez , I. J. Wang , and G. Velo‐Antón . 2017. “Trapped Within the City: Integrating Demography, Time Since Isolation and Population‐Specific Traits to Assess the Genetic Effects of Urbanization.” Molecular Ecology 26: 1498–1514. 10.1111/mec.14019. 28099779

[ece373372-bib-0092] Lowry, D. B. , S. Hoban , J. L. Kelley , et al. 2017. “Breaking RAD: An Evaluation of the Utility of Restriction Site‐Associated DNA Sequencing for Genome Scans of Adaptation.” Molecular Ecology Resources 17: 142–152. 10.1111/1755-0998.12635. 27860289 PMC5446919

[ece373372-bib-0093] Luikart, G. , M. Kardos , B. K. Hand , et al. 2019. “Population Genomics: Advancing Understanding of Nature.” In Population Genomics: Concepts, Approaches and Applications, edited by O. P. Rajora , 3–79. Springer International Publishing.

[ece373372-bib-0094] Magle, S. B. , V. M. Hunt , M. Vernon , and K. R. Crooks . 2012. “Urban Wildlife Research: Past, Present, and Future.” Biological Conservation 155: 23–32. 10.1016/j.biocon.2012.06.018.

[ece373372-bib-0095] Mantel, N. 1967. “The Detection of Disease Clustering and a Generalized Regression Approach.” Cancer Research 27: 209–220. 6018555

[ece373372-bib-0096] Marchini, J. , B. Howie , S. Myers , G. McVean , and P. Donnelly . 2007. “A New Multipoint Method for Genome‐Wide Association Studies by Imputation of Genotypes.” Nature Genetics 39, no. 7: 906–913. 17572673 10.1038/ng2088

[ece373372-bib-0097] Marcus, J. , W. Ha , R. F. Barber , and J. Novembre . 2021. “Fast and Flexible Estimation of Effective Migration Surfaces.” eLife 10: e61927. 10.7554/eLife.61927. 34328078 PMC8324296

[ece373372-bib-0098] Mather, N. , S. M. Traves , and S. Y. W. Ho . 2020. “A Practical Introduction to Sequentially Markovian Coalescent Methods for Estimating Demographic History From Genomic Data.” Ecology and Evolution 10: 579–589. 10.1002/ece3.5888. 31988743 PMC6972798

[ece373372-bib-0099] Mazet, O. , W. Rodríguez , S. Grusea , S. Boitard , and L. Chikhi . 2016. “On the Importance of Being Structured: Instantaneous Coalescence Rates and Human Evolution—Lessons for Ancestral Population Size Inference?” Heredity 116: 362–371. 10.1038/hdy.2015.104. 26647653 PMC4806692

[ece373372-bib-0100] McKinney, M. L. 2006. “Urbanization as a Major Cause of Biotic Homogenization.” Biological Conservation 127: 247–260. 10.1016/j.biocon.2005.09.005.

[ece373372-bib-0101] McRae, B. H. 2006. “Isolation by Resistance.” Evolution 60: 1551–1561. 10.1111/j.0014-3820.2006.tb00500.x. 17017056

[ece373372-bib-0102] McRae, B. H. , and P. Beier . 2007. “Circuit Theory Predicts Gene Flow in Plant and Animal Populations.” Proceedings of the National Academy of Sciences 104: 19885–19890. 10.1073/pnas.0706568104. PMC2148392 18056641

[ece373372-bib-0103] Meek, P. D. , G.‐A. Ballard , and P. J. S. Fleming . 2012. “A Permanent Security Post for Camera Trapping.” Australian Mammalogy 35: 123–127. 10.1071/AM12014.

[ece373372-bib-0104] Meirmans, P. G. 2012. “The Trouble With Isolation by Distance.” Molecular Ecology 21: 2839–2846. 10.1111/j.1365-294X.2012.05578.x. 22574758

[ece373372-bib-0105] Miles, L. S. , E. J. Carlen , Z. Nassrullah , J. Munshi‐South , and M. T. J. Johnson . 2025. “No Detectable Effect of Urbanization on Genetic Drift or Gene Flow in Specialist Herbivorous Insects of Milkweed.” PLoS One 20: e0318956. 10.1371/journal.pone.0318956. 39951478 PMC11828359

[ece373372-bib-0106] Miles, L. S. , R. J. Dyer , and B. C. Verrelli . 2018. “Urban Hubs of Connectivity: Contrasting Patterns of Gene Flow Within and Among Cities in the Western Black Widow Spider.” Proceedings of the Royal Society B: Biological Sciences 285 no. 1884:20191224. 10.1098/rspb.2018.1224. PMC6111156 30068686

[ece373372-bib-0107] Miles, L. S. , B. C. Verrelli , R. Adams , et al. 2025. “Were Bed Bugs the First Urban Pest Insect? Genome‐Wide Patterns of Bed Bug Demography Mirror Global Human Expansion.” Biology Letters 21: 20250061. 10.1098/rsbl.2025.0061. 40425045 PMC12115845

[ece373372-bib-0108] Miller, J. M. , C. I. Cullingham , and R. M. Peery . 2020. “The Influence of a Priori Grouping on Inference of Genetic Clusters: Simulation Study and Literature Review of the DAPC Method.” Heredity 125: 269–280. 10.1038/s41437-020-0348-2. 32753664 PMC7553915

[ece373372-bib-0109] Mills, L. S. , J. J. Citta , K. P. Lair , M. K. Schwartz , and D. A. Tallmon . 2000. “Estimating Animal Abundance Using Noninvasive Dna Sampling: Promise and Pitfalls.” Ecological Applications 10: 283–294. 10.1890/1051-0761(2000)010[0283:EAAUND]2.0.CO;2.

[ece373372-bib-0110] Mueller, J. C. , M. Carrete , S. Boerno , H. Kuhl , J. L. Tella , and B. Kempenaers . 2020. “Genes Acting in Synapses and Neuron Projections Are Early Targets of Selection During Urban Colonization.” Molecular Ecology 29: 3403–3412. 32310323 10.1111/mec.15451

[ece373372-bib-0111] Mueller, J. C. , H. Kuhl , S. Boerno , J. L. Tella , M. Carrete , and B. Kempenaers . 2018. “Evolution of Genomic Variation in the Burrowing Owl in Response to Recent Colonization of Urban Areas.” Proceedings of the Royal Society B: Biological Sciences 285: 20180206. 10.1098/rspb.2018.0206. PMC5966595 29769357

[ece373372-bib-0112] Mueller, J. C. , J. Partecke , B. J. Hatchwell , K. J. Gaston , and K. L. Evans . 2013. “Candidate Gene Polymorphisms for Behavioural Adaptations During Urbanization in Blackbirds.” Molecular Ecology 22: 3629–3637. 10.1111/mec.12288. 23495914

[ece373372-bib-0113] Munshi‐South, J. 2012. “Urban Landscape Genetics: Canopy Cover Predicts Gene Flow Between White‐Footed Mouse ( *Peromyscus leucopus* ) Populations in New York City.” Molecular Ecology 21: 1360–1378. 10.1111/j.1365-294X.2012.05476.x. 22320856

[ece373372-bib-0114] Munshi‐South, J. , and K. Kharchenko . 2010. “Rapid, Pervasive Genetic Differentiation of Urban White‐Footed Mouse ( *Peromyscus leucopus* ) Populations in New York City.” Molecular Ecology 19: 4242–4254. 10.1111/j.1365-294X.2010.04816.x. 20819163

[ece373372-bib-0115] Munshi‐South, J. , C. P. Zolnik , and S. E. Harris . 2016. “Population Genomics of the Anthropocene: Urbanization Is Negatively Associated With Genome‐Wide Variation in White‐Footed Mouse Populations.” Evolutionary Applications 9: 546–564. 10.1111/eva.12357. 27099621 PMC4831458

[ece373372-bib-0116] Narum, S. R. , and J. E. Hess . 2011. “Comparison of FST Outlier Tests for SNP Loci Under Selection.” Molecular Ecology Resources 11: 184–194. 10.1111/j.1755-0998.2011.02987.x. 21429174

[ece373372-bib-0117] Nazareno, A. G. , J. B. Bemmels , C. W. Dick , and L. G. Lohmann . 2017. “Minimum Sample Sizes for Population Genomics: An Empirical Study From an Amazonian Plant Species.” Molecular Ecology Resources 17: 1136–1147. 10.1111/1755-0998.12654. 28078808

[ece373372-bib-0118] Nei, M. 1987. “Molecular Evolutionary Genetics.” In Molecular Evolutionary Genetics. Columbia University Press.

[ece373372-bib-0119] Novembre, J. , and M. Stephens . 2008. “Interpreting Principal Component Analyses of Spatial Population Genetic Variation.” Nature Genetics 40: 646–649. 10.1038/ng.139. 18425127 PMC3989108

[ece373372-bib-0120] Oden, N. L. , and R. R. Sokal . 1986. “Directional Autocorrelation: An Extension of Spatial Correlograms to Two Dimensions.” Systematic Zoology 35: 608–617.

[ece373372-bib-0121] Palumbi, S. , A. Martin , S. Romano , W. O. McMillan , L. Stice , and G. Grabowski . 2002. The Simple Fool's Guide to PCR, Version 2.0. University of Hawaii.

[ece373372-bib-0122] Parsons, K. M. , M. Everett , M. Dahlheim , and L. Park . 2018. “Water, Water Everywhere: Environmental DNA Can Unlock Population Structure in Elusive Marine Species.” Royal Society Open Science 5: 180537. 10.1098/rsos.180537. 30225045 PMC6124077

[ece373372-bib-0123] Penone, C. , C. Kerbiriou , J.‐F. Julien , et al. 2013. “Urbanisation Effect on Orthoptera: Which Scale Matters?” Insect Conservation and Diversity 6: 319–327. 10.1111/j.1752-4598.2012.00217.x.

[ece373372-bib-0124] Peterman, W. E. 2018. “ResistanceGA: An R Package for the Optimization of Resistance Surfaces Using Genetic Algorithms.” Methods in Ecology and Evolution 9: 1638–1647. 10.1111/2041-210X.12984.

[ece373372-bib-0125] Peterson, B. K. , J. N. Weber , E. H. Kay , H. S. Fisher , and H. E. Hoekstra . 2012. “Double Digest RADseq: An Inexpensive Method for De Novo SNP Discovery and Genotyping in Model and Non‐Model Species.” PLoS One 7, no. 5: e37135. 10.1371/journal.pone.0037135. 22675423 PMC3365034

[ece373372-bib-0126] Petkova, D. , J. Novembre , and M. Stephens . 2016. “Visualizing Spatial Population Structure With Estimated Effective Migration Surfaces.” Nature Genetics 48: 94–103. 10.1101/011809. 26642242 PMC4696895

[ece373372-bib-0127] Pritchard, J. K. , M. Stephens , and P. Donnelly . 2000. “Inference of Population Structure Using Multilocus Genotype Data.” Genetics 155: 945–959. 10.1093/genetics/155.2.945. 10835412 PMC1461096

[ece373372-bib-0128] Puckett, E. E. 2016. “Variability in Total Project and Per Sample Genotyping Costs Under Varying Study Designs Including With Microsatellites or SNPs to Answer Conservation Genetic Questions.” Conservation Genetics Resources 0: 0. 10.1007/s12686-016-0643-7.

[ece373372-bib-0129] Puckett, E. E. , J. Park , M. Combs , et al. 2016. “Global Population Divergence and Admixture of the Brown Rat ( *Rattus norvegicus* ).” Proceedings of the Royal Society B: Biological Sciences 283: 20161762. 10.1098/rspb.2016.1762. PMC5095384 27798305

[ece373372-bib-0130] Raj, A. , M. Stephens , and J. K. Pritchard . 2014. “FastSTRUCTURE: Variational Inference of Population Structure in Large SNP Data Sets.” Genetics 197: 573–589. 10.1534/genetics.114.164350. 24700103 PMC4063916

[ece373372-bib-0131] Rajora, O. P. , ed. 2024. Population Genomics: Crop Plants. Springer International Publishing.

[ece373372-bib-0132] Raxworthy, C. J. , and B. T. Smith . 2021. “Mining Museums for Historical DNA: Advances and Challenges in Museomics.” Trends in Ecology & Evolution 36: 1049–1060. 10.1016/j.tree.2021.07.009. 34456066

[ece373372-bib-0133] Reiner, G. , M. Lang , and H. Willems . 2019. “Impact of Different Panels of Microsatellite Loci, Different Numbers of Loci, Sample Sizes, and Gender Ratios on Population Genetic Results in Red Deer.” European Journal of Wildlife Research 65: 25. 10.1007/s10344-019-1262-x.

[ece373372-bib-0134] Rellstab, C. , F. Gugerli , A. J. Eckert , A. M. Hancock , and R. Holderegger . 2015. “A Practical Guide to Environmental Association Analysis in Landscape Genomics.” Molecular Ecology 24: 4348–4370. 10.1111/mec.13322. 26184487

[ece373372-bib-0135] Richardson, J. L. , M. K. Burak , C. Hernandez , et al. 2017. “Using Fine‐Scale Spatial Genetics of Norway Rats to Improve Control Efforts and Reduce Leptospirosis Risk in Urban Slum Environments.” Evolutionary Applications 10: 323–337. 10.1111/eva.12449. 28352293 PMC5367079

[ece373372-bib-0136] Richmond, J. Q. , D. A. Wood , M. F. Westphal , et al. 2017. “Persistence of Historical Population Structure in an Endangered Species Despite Near‐Complete Biome Conversion in California's San Joaquin Desert.” Molecular Ecology 26: 3618–3635. 10.1111/mec.14125. 28370723

[ece373372-bib-0137] Rocha, J. J. , S. A. Jayaram , T. J. Stevens , et al. 2023. “Functional Unknomics: Systematic Screening of Conserved Genes of Unknown Function.” PLoS Biology 21, no. 8: e3002222. 37552676 10.1371/journal.pbio.3002222 PMC10409296

[ece373372-bib-0138] Sabeti, P. C. , D. E. Reich , J. M. Higgins , et al. 2002. “Detecting Recent Positive Selection in the Human Genome From Haplotype Structure.” Nature 419, no. 6909: 832–837. 12397357 10.1038/nature01140

[ece373372-bib-0139] Saenz, V. L. , W. Booth , C. Schal , and E. L. Vargo . 2012. “Genetic Analysis of Bed Bug Populations Reveals Small Propagule Size Within Individual Infestations but High Genetic Diversity Across Infestations From the Eastern United States.” Journal of Medical Entomology 49: 865–875. 10.1603/ME11202. 22897047

[ece373372-bib-0140] Santangelo, J. S. , M. T. Johnson , and R. W. Ness . 2025. “Signatures of Selective Sweeps in Urban and Rural White Clover Populations.” Evolution 79, no. 10: 2115–2132. 10.1093/evolut/qpaf138. 40623003

[ece373372-bib-0141] Santangelo, J. S. , R. W. Ness , B. Cohan , et al. 2022. “Global Urban Environmental Change Drives Adaptation in White Clover.” Science 375, no. 6586: 1275–1281. 35298255 10.1126/science.abk0989

[ece373372-bib-0142] Santiago, E. , I. Novo , A. F. Pardiñas , M. Saura , J. Wang , and A. Caballero . 2020. “Recent Demographic History Inferred by High‐Resolution Analysis of Linkage Disequilibrium.” Molecular Biology and Evolution 37: 3642–3653. 10.1093/molbev/msaa169. 32642779

[ece373372-bib-0143] Satam, H. , K. Joshi , U. Mangrolia , et al. 2023. “Next‐Generation Sequencing Technology: Current Trends and Advancements.” Biology 12: 997. 10.3390/biology12070997. 37508427 PMC10376292

[ece373372-bib-0144] Schmidt, C. , and C. Garroway . 2022. “Systemic Racism Alters Wildlife Genetic Diversity.” Proceedings of the National Academy of Sciences 119: e2102860119. 10.32942/osf.io/wbq83. PMC9618126 36256811

[ece373372-bib-0145] Schmitz, G. , A. Linstädter , A. S. Frank , et al. 2024. “Environmental Filtering of Life‐History Trait Diversity in Urban Populations of *Arabidopsis thaliana* .” Journal of Ecology 112, no. 1: 14–27. 10.1111/1365-2745.14211.

[ece373372-bib-0147] Schultz, A. J. B. J. , K. C. Adams , W. B. Bell , G. B. P. Ludt , and J. E. Vendetti . 2021. “Natural History Collections are Critical Resources for Contemporary and Future Studies of Urban Evolution.” Evolutionary Applications 14: 233–247. 10.1111/eva.13045. 33519967 PMC7819571

[ece373372-bib-0146] Sham, P. C. , and S. M. Purcell . 2014. “Statistical Power and Significance Testing in Large‐Scale Genetic Studies.” Nature Reviews. Genetics 15: 335–346. 10.1038/nrg3706. 24739678

[ece373372-bib-0148] Sigsgaard, E. E. , I. B. Nielsen , S. S. Bach , et al. 2016. “Population Characteristics of a Large Whale Shark Aggregation Inferred From Seawater Environmental DNA.” Nature Ecology & Evolution 1: 1–5. 10.1038/s41559-016-0004. 28812572

[ece373372-bib-0149] Sih, A. , M. C. O. Ferrari , and D. J. Harris . 2011. “Evolution and Behavioural Responses to Human‐Induced Rapid Environmental Change.” Evolutionary Applications 4: 367–387. 10.1111/j.1752-4571.2010.00166.x. 25567979 PMC3352552

[ece373372-bib-0150] Sih, A. , B. G. Jonsson , and G. Luikart . 2000. “Habitat Loss: Ecological, Evolutionary and Genetic Consequences.” Trends in Ecology & Evolution 15: 132–134. 10.1016/S0169-5347(99)01799-1.

[ece373372-bib-0151] Smouse, P. E. , S. C. Banks , and R. Peakall . 2017. “Converting Quadratic Entropy to Diversity: Both Animals and Alleles Are Diverse, but Some Are More Diverse Than Others.” PLoS One 12: e0185499. 10.1371/journal.pone.0185499. 29088229 PMC5663342

[ece373372-bib-0152] Sokal, R. R. 1986. “Spatial Data Analysis and Historical Processes.” In Data Analysis and Informatics, IV, edited by E. Diday , 29–43. North‐Holland.

[ece373372-bib-0153] Sokal, R. R. , and F. J. Rohlf . 1995. Biometry. 3rd ed, 813–819. Freeman ISBN 0‐7167‐2411‐1.

[ece373372-bib-0154] Soni, V. , and J. D. Jensen . 2024. “Temporal Challenges in Detecting Balancing Selection From Population Genomic Data.” G3: Genes, Genomes, Genetics 14: jkae069. 10.1093/g3journal/jkae069. 38551137 PMC11152078

[ece373372-bib-0155] Stephan, W. 2019. “Selective Sweeps.” Genetics 211, no. 1: 5–13. 10.1534/genetics.118.301319. 30626638 PMC6325696

[ece373372-bib-0156] Stockwell, C. A. , A. P. Hendry , and M. T. Kinnison . 2003. “Contemporary Evolution Meets Conservation Biology.” Trends in Ecology & Evolution 18: 94–101. 10.1016/S0169-5347(02)00044-7.

[ece373372-bib-0157] Sunde, J. , Y. Yıldırım , P. Tibblin , and A. Forsman . 2020. “Comparing the Performance of Microsatellites and RADseq in Population Genetic Studies: Analysis of Data for Pike ( *Esox lucius* ) and a Synthesis of Previous Studies.” Frontiers in Genetics 11: 218. 10.3389/fgene.2020.00218. 32231687 PMC7082332

[ece373372-bib-0158] Supple, M. A. , and B. Shapiro . 2018. “Conservation of Biodiversity in the Genomics Era.” Genome Biology 19: 131. 10.1186/s13059-018-1520-3. 30205843 PMC6131752

[ece373372-bib-0182] Szpiech, Z. A. , and R. D. Hernandez . 2014. “selscan: An Efficient Multithreaded Program to Perform EHH‐Based Scans for Positive Selection.” Molecular Biology and Evolution 31, no. 10: 2824–2827. 10.1093/molbev/msu211. 25015648 PMC4166924

[ece373372-bib-0159] Tang, Q. , E. L. Vargo , I. Ahmad , et al. 2024. “Solving the 250‐Year Old Mystery of the Origin and Spread of the German Cockroach, *Blattella germanica* .” Proceedings of the National Academy of Sciences of the United States of America 121: w2401185121. 10.1073/pnas.2401185121. PMC11145273 38768340

[ece373372-bib-0160] Thioulouse, J. , S. Dray , A. Dufour , A. Siberchicot , T. Jombart , and S. Pavoine . 2018. Multivariate Analysis of Ecological Data With ade4. Springer. 10.1007/978-1-4939-8850-1.

[ece373372-bib-0161] Thompson, K. A. , M. Renaudin , and M. T. J. Johnson . 2016. “Urbanization Drives the Evolution of Parallel Clines in Plant Populations.” Proceedings of the Royal Society B: Biological Sciences 283: 20162180. 10.1098/rspb.2016.2180. PMC5204167 28003451

[ece373372-bib-0162] United Nations . 2014. World Urbanization Prospects: The 2014 Revision, Highlights.

[ece373372-bib-0163] United Nations . 2019. Decade on Ecosystem Restoration (2021‐2023), Highlights.

[ece373372-bib-0164] Vieira, M. L. C. , L. Santini , A. L. Diniz , and C. d. F. Munhoz . 2016. “Microsatellite Markers: What They Mean and Why They Are So Useful.” Genetics and Molecular Biology 39: 312–328. 10.1590/1678-4685-GMB-2016-0027. 27561112 PMC5004837

[ece373372-bib-0165] Voight, B. F. , S. Kudaravalli , X. Wen , and J. K. Pritchard . 2006. “A Map of Recent Positive Selection in the Human Genome.” PLoS Biology 4, no. 3: e72. 16494531 10.1371/journal.pbio.0040072 PMC1382018

[ece373372-bib-0166] von Thaden, A. , C. Nowak , A. Tiesmeyer , et al. 2020. “Applying Genomic Data in Wildlife Monitoring: Development Guidelines for Genotyping Degraded Samples With Reduced Single Nucleotide Polymorphism Panels.” Molecular Ecology Resources 20: 662–680. 10.1111/1755-0998.13136. PMC7199164 31925943

[ece373372-bib-0167] Wallace, J. G. , and S. E. Mitchell . 2017. “Genotyping‐By‐Sequencing.” Current Protocols in Plant Biology 2, no. 1: 64–77. 31725977 10.1002/cppb.20042

[ece373372-bib-0168] Wang, J. 2005. “Estimation of Effective Population Sizes From Data on Genetic Markers.” Philosophical Transactions of the Royal Society, B: Biological Sciences 360: 1395–1409. 10.1098/rstb.2005.1682. PMC1847562 16048783

[ece373372-bib-0169] Weir, B. S. , and C. C. Cockerham . 1984. “Estimating F‐Statistics for the Analysis of Population Structure.” Evolution 38: 1358–1370. 28563791 10.1111/j.1558-5646.1984.tb05657.x

[ece373372-bib-0170] Willoughby, J. R. , J. A. Ivy , R. C. Lacy , J. M. Doyle , and J. A. DeWoody . 2017. “Inbreeding and Selection Shape Genomic Diversity in Captive Populations: Implications for the Conservation of Endangered Species.” PLoS One 12: e0175996. 10.1371/journal.pone.0175996. 28423000 PMC5396937

[ece373372-bib-0171] Winchell, K. M. , K. J. Aviles‐Rodriguez , E. J. Carlen , et al. 2022. “Moving Past the Challenges and Misconceptions in Urban Adaptation Research.” Ecology and Evolution 12: e9552. 36425909 10.1002/ece3.9552 PMC9679025

[ece373372-bib-0172] Winiarski, K. J. , W. E. Peterman , and K. McGarigal . 2020. “Evaluation of the R Package ‘Resistancega’: A Promising Approach Towards the Accurate Optimization of Landscape Resistance Surfaces.” Molecular Ecology Resources 20: 1583–1596. 10.1111/1755-0998.13217. 32608130

[ece373372-bib-0173] Wood, D. A. , J. P. Rose , B. J. Halstead , R. E. Stoelting , K. E. Swaim , and A. G. Vandergast . 2020. “Combining Genetic and Demographic Monitoring Better Informs Conservation of an Endangered Urban Snake.” PLoS One 15: e0231744. 10.1371/journal.pone.0231744. 32369486 PMC7200000

[ece373372-bib-0174] Wright, A. L. , J. R. Anson , V. Leo , et al. 2022. “Urban Restoration of Common Species: Population Genetics of Reintroduced Native Brush Rats *Rattus fuscipes* in Sydney, Australia.” Animal Conservation 25: 825–836. 10.1111/acv.12787.

[ece373372-bib-0175] Wright, S. 1943. “Isolation by Distance.” Genetics 28: 114–138. 10.1093/genetics/28.2.114. 17247074 PMC1209196

[ece373372-bib-0176] Wright, S. 1965. “The Interpretation of Population Structure by F‐Statistics With Special Regard to Systems of Mating.” Evolution 19: 395–420. 10.2307/2406450.

[ece373372-bib-0177] Xuereb, A. , L. Benestan , É. Normandeau , et al. 2018. “Asymmetric Oceanographic Processes Mediate Connectivity and Population Genetic Structure, as Revealed by RADseq, in a Highly Dispersive Marine Invertebrate ( *Parastichopus californicus* ).” Molecular Ecology 27: 2347–2364. 10.1111/mec.14589. 29654703

[ece373372-bib-0178] Yakub, M. , and P. Tiffin . 2017. “Living in the City: Urban Environments Shape the Evolution of a Native Annual Plant.” Global Change Biology 23, no. 5: 2082–2089. 27718531 10.1111/gcb.13528

[ece373372-bib-0179] Zane, L. , L. Bargelloni , and T. Patarnello . 2002. “Strategies for Microsatellite Isolation: A Review.” Molecular Ecology 11: 1–16. 10.1046/j.0962-1083.2001.01418.x. 11903900

[ece373372-bib-0180] Zhivotovsky, L. A. 2015. “Relationships Between Wright's FST and FIS Statistics in a Context of Wahlund Effect.” Journal of Heredity 106: 306–309. 10.1093/jhered/esv019. 25888609

[ece373372-bib-0181] Zhu, F. , L. Lavine , S. O'Neal , et al. 2016. “Insecticide Resistance and Management Strategies in Urban Ecosystems.” Insects 7: 2. 10.3390/insects7010002. 26751480 PMC4808782

