# Supplementary material for: A Beginner's How‐To Guide to Urban Population Genetics and Genomics
Source: Ecol Evol. 2026 Apr 7;16(4):e73372. doi: 10.1002/ece3.73372 (PMC13054242; doi:10.1002/ece3.73372)
Supplement: Supplementary file 1 — Data S1: ece373372‐sup‐0001‐supinfo.docx. [file ECE3-16-e73372-s001.docx]

# A Beginner’s Guide to Urban Population Genetics and Genomics

Elizabeth Carlen, Lindsay Miles, Kevin Aviles-Rodriguez, Warren Booth

2025-03-11

## Load Required Libraries

library(ggplot2)

## Warning: package 'ggplot2' was built under R version 4.3.1

library(adegenet)

## Loading required package: ade4

##
## /// adegenet 2.1.10 is loaded ////////////
##
## > overview: '?adegenet'
## > tutorials/doc/questions: 'adegenetWeb()'
## > bug reports/feature requests: adegenetIssues()

library(hierfstat)

##
## Attaching package: 'hierfstat'

## The following objects are masked from 'package:adegenet':
##
## Hs, read.fstat

library(ade4)
library(ecodist)

## Warning: package 'ecodist' was built under R version 4.3.1

library(colorspace)

## Warning: package 'colorspace' was built under R version 4.3.3

library(pcadapt)

## Warning: package 'pcadapt' was built under R version 4.3.3

*#install.packages("devtools")*
*#library("devtools")*
*#install_github("jdstorey/qvalue")*
library(vcfR)

## Warning: package 'vcfR' was built under R version 4.3.1

##
## ***** *** vcfR *** *****
## This is vcfR 1.15.0
## browseVignettes('vcfR') # Documentation
## citation('vcfR') # Citation
## ***** ***** ***** *****

*#install_github("whitlock/OutFLANK")*
library("OutFLANK")

## Loading required package: qvalue

library(qqman)

##

## For example usage please run: vignette('qqman')

##

## Citation appreciated but not required:

## Turner, (2018). qqman: an R package for visualizing GWAS results using Q-Q and manhattan plots. Journal of Open Source Software, 3(25), 731, https://doi.org/10.21105/joss.00731.

##

## Set Working Directory

*#setwd("/Users/ecarlen/Desktop/manuscripts/how to urban pop gen/Sim Data")*
population_colors<-population_colors <- c("A" = "#496849", "B" = "#654783")

## Read Genetic Data

Our genetic data was based on a simulation using Hudson (2002) ms program. We simulated two populations with equal migration between them and 1000 segregating sites:

./ms 40 1 -s 1000 -I 2 20 20 -m 1 2 2.5 -m 2 1 2.5

The raw file ms_2pop_eqmig_stdout.txt is the standard output from the program. We have manually edited the output into a structure file format that can then be loaded as a genind object for the adegenet program.

genetic_data <- read.structure("test.stru", n.ind = 20, n.loc = 1000, col.lab = 1, col.pop = 2, onerowperind = FALSE,row.marknames=1, quiet=TRUE )

##
## Which other optional columns should be read (press 'return' when done)?

genetic_data

## /// GENIND OBJECT /////////
##
## // 20 individuals; 1,000 loci; 2,000 alleles; size: 667 Kb
##
## // Basic content
## @tab: 20 x 2000 matrix of allele counts
## @loc.n.all: number of alleles per locus (range: 2-2)
## @loc.fac: locus factor for the 2000 columns of @tab
## @all.names: list of allele names for each locus
## @ploidy: ploidy of each individual (range: 2-2)
## @type: codom
## @call: read.structure(file = "test.stru", n.ind = 20, n.loc = 1000,
## onerowperind = FALSE, col.lab = 1, col.pop = 2, row.marknames = 1,
## quiet = TRUE)
##
## // Optional content
## @pop: population of each individual (group size range: 10-10)

## Compute Genetic Distance Matrix

geneticdistance <- dist(genetic_data, method = "euclidean")

## Load and Process Geographic Data

We simulated latitude and longitude data for these simulated samples. This is because many of the programs mentioned in the text require location data.

location_data <- data.frame(
 sample_ID = paste0("ind", 1:20),
 Latitude = c(39.8745, 40.4507, 40.232, 40.0987, 39.656, 39.656, 39.5581, 40.3662, 40.1011, 40.2081,
 41.1119, 40.6395, 40.7921, 40.8664, 40.9561, 41.2852, 40.6997, 41.0142, 41.0924, 40.5465),
 Longitude = c(-100.4794, -99.5301, -99.6676, -100.2877, -100.3182, -100.3166, -100.1958, -99.9752, -100.0681, -100.2088,
 -100.8925, -101.3295, -101.4349, -100.5511, -100.5344, -100.6916, -101.1954, -101.4023, -100.8158, -101.0598)
)
location_data$population <- ifelse(1:20 <= 10, "A", "B")

## Compute Geographic Distance Matrix

geographicdistance <- dist(location_data[,2:3])

## Plot Sample Locations

location_map <- ggplot(location_data, aes(x = Longitude, y = Latitude, color = population)) +
 geom_point(size = 7) +
 scale_color_manual(values = c("#496849", "#654783")) +
 theme_minimal() +
 theme(panel.grid = element_blank(), axis.line = element_line(color = "black"))
location_map


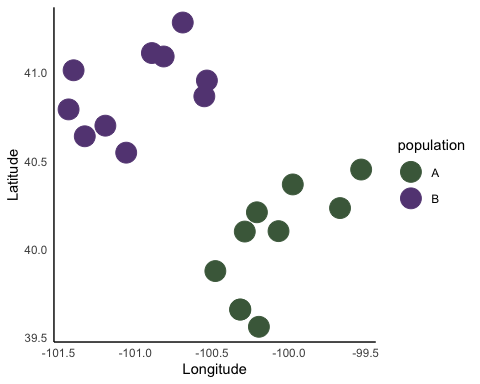


## Compute FST

We calculate both Weir & Cockerham’s Fst and pairwise Fst

hierfstat_data <- genind2hierfstat(genetic_data)
fst_result <- wc(hierfstat_data)
fst_result

## $FST
## [1] 0.4733827
##
## $FIS
## [1] 0.05996132

pairwise_fst <- pairwise.WCfst(hierfstat_data)
pairwise_fst

## A B
## A NA 0.4733827
## B 0.4733827 NA

## Mantel Test: Isolation by Distance

We are running this on an individual basis. Anthor option when you have more than two sampled populations is to run this with Fst pairwise genetic distance and the euclidean distance measured between sample population locations.

mantel_test <- mantel.rtest(as.dist(geneticdistance), as.dist(geographicdistance), nrepet = 999)
regression_mantel_test <- lm(as.vector(as.dist(geneticdistance)) ~ as.vector(as.dist(geographicdistance)))
summary(regression_mantel_test)

##
## Call:
## lm(formula = as.vector(as.dist(geneticdistance)) ~ as.vector(as.dist(geographicdistance)))
##
## Residuals:
## Min 1Q Median 3Q Max
## -34.274 -12.779 -1.854 13.058 41.479
##
## Coefficients:
## Estimate Std. Error t value Pr(>|t|)
## (Intercept) 18.957 2.792 6.79 1.43e-10 ***
## as.vector(as.dist(geographicdistance)) 17.586 2.586 6.80 1.35e-10 ***
## ---
## Signif. codes: 0 '***' 0.001 '**' 0.01 '*' 0.05 '.' 0.1 ' ' 1
##
## Residual standard error: 17.32 on 188 degrees of freedom
## Multiple R-squared: 0.1974, Adjusted R-squared: 0.1931
## F-statistic: 46.23 on 1 and 188 DF, p-value: 1.354e-10

## Mantel Correlogram

mantel_correlogram <- mgram(geneticdistance, geographicdistance, stepsize = 0.1, nperm = 1000, nboot = 500, pboot = 0.9, cboot = 0.95)
mantel_correlogram_dataframe <- as.data.frame(mantel_correlogram$mgram)
mantel_correlogram_dataframe$significance <- ifelse(mantel_correlogram_dataframe$pval < 0.05, "Significant", "Not Significant")

## Plot Mantel Correlogram

ggplot(mantel_correlogram_dataframe, aes(x = lag, y = mantelr)) +
 geom_line(color = "black") +
 geom_point(aes(shape = significance, fill = significance), size = 3, color = "black") +
 geom_hline(yintercept = 0, linetype = "dashed", color = "gray") +
 scale_shape_manual(values = c(21, 1)) +
 scale_fill_manual(values = c("black", "white")) +
 theme_classic() +
 labs(title = "Mantel Correlogram", x = "Distance Class (Lag)", y = "Mantel r") +
 theme(legend.title = element_blank())


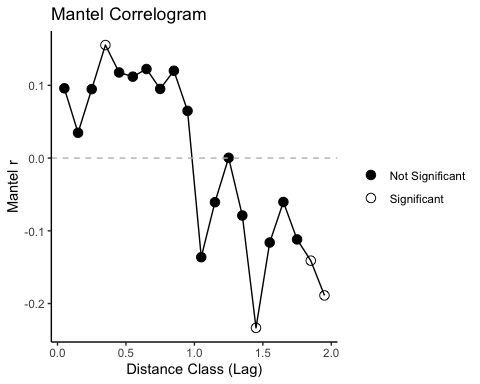


## Principal Component Analysis (PCA)

PCA is model-free and assumption-free. This can be used to identify putative population structure with and without *a priori* population assignment.

PCA <- dudi.pca(genetic_data, cent = TRUE, scale = FALSE, scannf = FALSE, nf = 10)
PCA_variance <- PCA$eig / sum(PCA$eig) * 100
PCA_dataframe <- data.frame(
 SampleID = rownames(PCA$li),
 PC1 = PCA$li[, 1],
 PC2 = PCA$li[, 2],
 Population = location_data$population
)

## Plot PCA

PCA_plot <- ggplot(PCA_dataframe, aes(x = PC1, y = PC2, col= Population)) +
 geom_jitter(width = 1, height = 0.5, alpha = 0.7, size=3) +
 scale_color_manual(values = c("A" = "#496849", "B" = "#654783")) +
 theme_classic()
PCA_plot


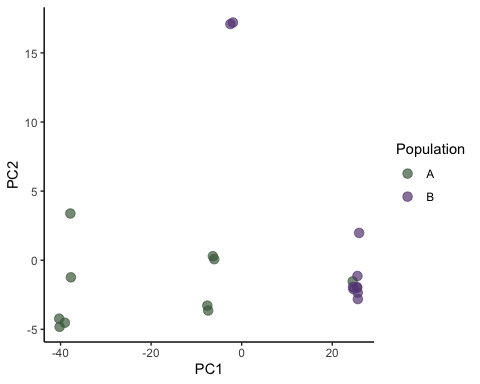


## Discriminant Analysis of Principal Components (DAPC)

DAPC requires groups to be defined, but some functions within adegenet can find the optimal number of clusters.

genetic_groups <- find.clusters(genetic_data, n.clust=10)


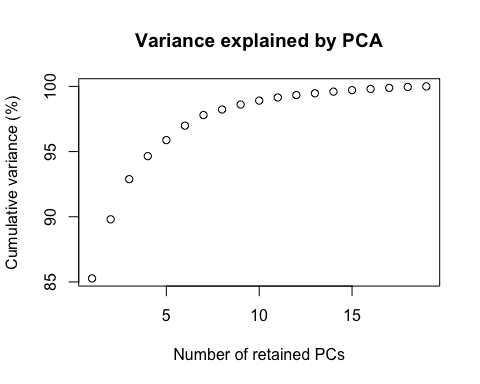


## Choose the number PCs to retain (>= 1):

dapc_optimal <- dapc(genetic_data, var.contrib=TRUE, scale=TRUE, n.pca=3, n.da=1)
scatter(dapc_optimal, scree.da=FALSE, cstar=0, cex=1.5, clab=0, leg=TRUE, col=c("#496849", "#654783"))


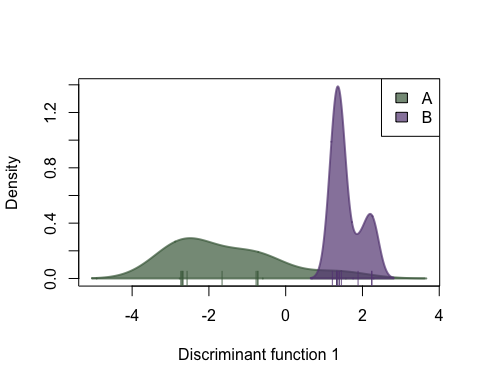


Because the optimum PCs=1 (e.g., single discriminant function, *k* = 2), the DAPC only has the one discriminant function to graph. Most data types will have more than the one and the DAPC will look similar to a PCA.

We can run a STRUCTURE-like plot as well:

compoplot(dapc_optimal, lab="", ncol=1, xlab="individuals", col=population_colors)


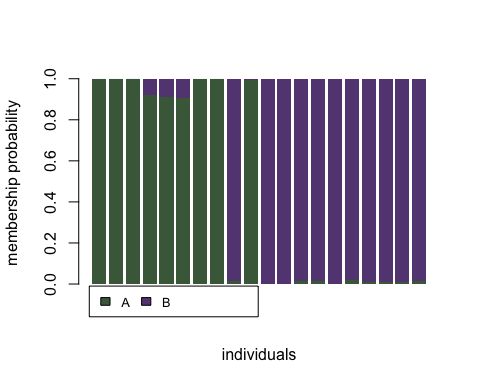


Here we can see that individuals are not assigned 100% into cluster groups. We expect this based on our simulation that allowed for migration between the two populations.

***## Converting Genind to Gtypes***
snps_gtypes = genind2genpop(genetic_data)

##
## Converting data from a genind to a genpop object...
##
## ...done.

##Spatial Principal COmponent Analysis (SPCA) First add Lat/Long to the genind object, then run spca…

genetic_data$other$xy<-location_data[,2:3]
myspca<-spca(genetic_data, ask=FALSE, type=1, scannf=FALSE)

## Registered S3 method overwritten by 'proxy':
## method from
## dim.dist ecodist

## Registered S3 method overwritten by 'spdep':
## method from
## plot.mst ape

## Registered S3 methods overwritten by 'adegraphics':
## method from
## biplot.dudi ade4
## kplot.foucart ade4
## kplot.mcoa ade4
## kplot.mfa ade4
## kplot.pta ade4
## kplot.sepan ade4
## kplot.statis ade4
## scatter.coa ade4
## scatter.dudi ade4
## scatter.nipals ade4
## scatter.pco ade4
## score.acm ade4
## score.mix ade4
## score.pca ade4
## screeplot.dudi ade4

## Registered S3 methods overwritten by 'adespatial':
## method from
## plot.multispati adegraphics
## print.multispati ade4
## summary.multispati ade4


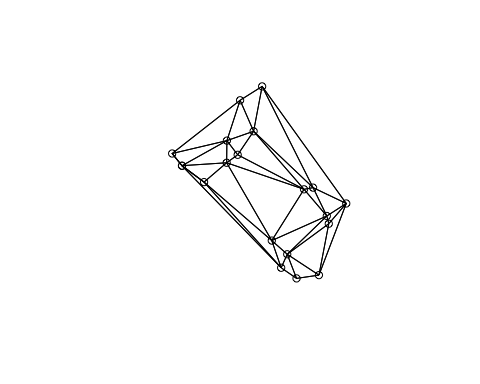


barplot(myspca$eig, main="A variant of the plot\n of sPCA eigenvalues", col=spectral(length(myspca$eig)))
legend("topright", fill=spectral(2), leg=c("Global structures", "Local structures"))
abline(h=0,col="grey")


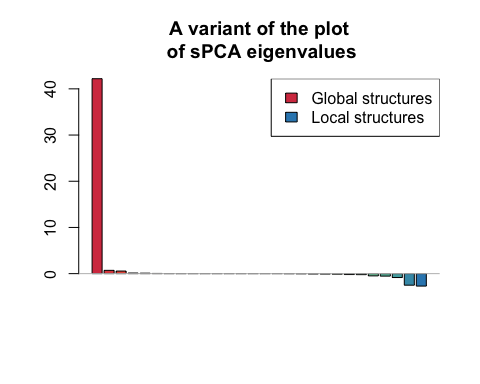


screeplot.spca(myspca)


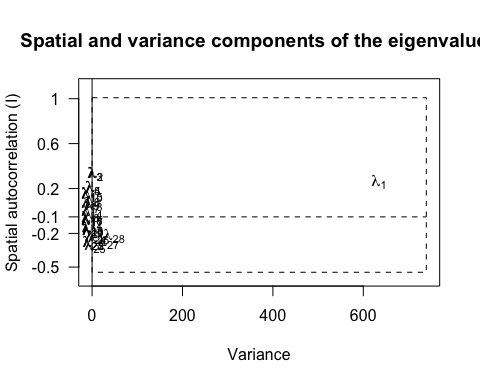


plot(myspca)


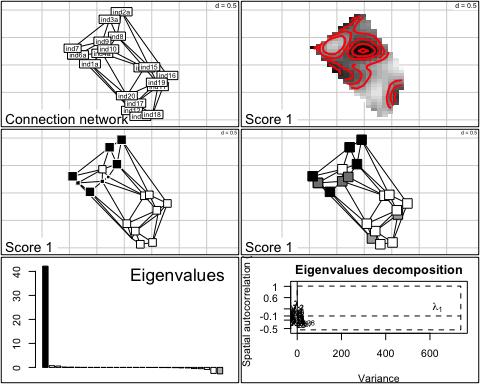


Let’s look at Interpolating principal components to get the map of genetic clines

library(akima)
x <- other(genetic_data)$xy[,1]
y <- other(genetic_data)$xy[,2]
temp <- interp(x, y, myspca$li[,1])
image(temp, col=azur(100))
points(x,y)


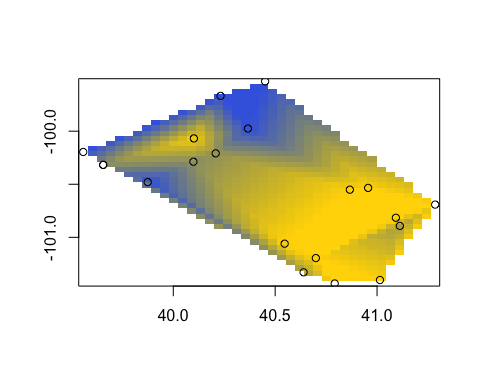


interpX <- seq(min(x),max(x),le=200)
interpY <- seq(min(y),max(y),le=200)
temp <- interp(x, y, myspca$ls[,1], xo=interpX, yo=interpY)
image(temp, col=azur(100))
points(x,y)


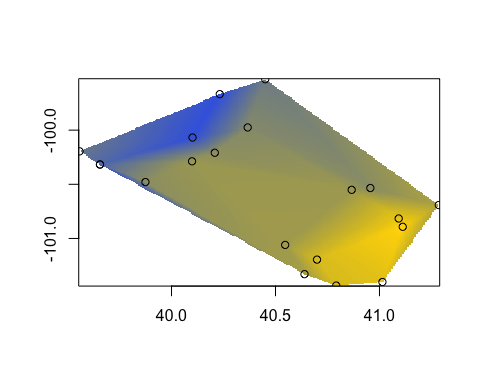


spca_col<-rainbow_hcl(28)
colorplot(genetic_data$other$xy,myspca$ls, axes=1:2, transp=FALSE, add=FALSE, cex=3, col=spca_col)


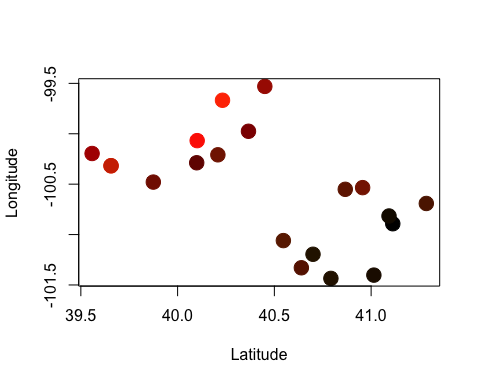


## Adaptive genetic Variation

# Outlier tests

For this section we will use subsets of publicly available data from <https://raw.githubusercontent.com/BayLab/MarineGenomicsData/main/week7.tar.gz>

path_to_file <- "./filtered_3699snps_californicus.vcf"
calidata <- read.pcadapt(path_to_file, type = "vcf")

## Warning in file2other(input, type, match.arg(type.out), match.arg(allele.sep)):
## Converter vcf to pcadapt is deprecated. Please use PLINK for conversion to bed
## (and QC).

## No variant got discarded.
## Summary:
##
## - input file: ./filtered_3699snps_californicus.vcf
## - output file: /var/folders/nr/33856cdn7b7cpbsrch43j0sh0000gp/T//RtmplaeBpP/filee4f12caa57d2.pcadapt
##
## - number of individuals detected: 717
## - number of loci detected: 3699
##
## 3699 lines detected.
## 717 columns detected.

meta <- read.csv("californicus_metadata.csv")

We will look at the SNP data using a Manhattan plot and test which values are significant

x <- pcadapt(input=calidata,K=5)
plot(x, options="manhattan")


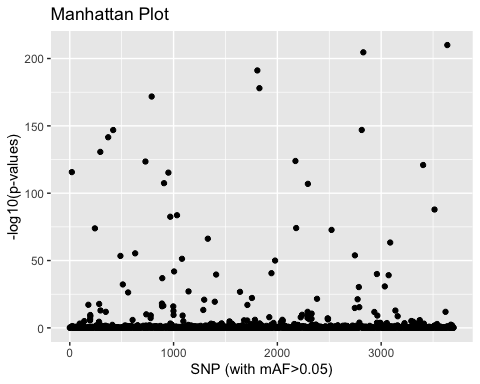


qval <- qvalue(x$pvalues)$qvalues
outliers <- which(qval<0.1)
length(outliers)

## [1] 143

Ok, so There are 143 outliers that in the SNP data. You can go back and see which SNPs are outliers based on the vector “outliers” that was generated. Then, if your vcf file containes annotations, you can manually check to see if these SNPs are in gene regions.

We can also detect outlier FSTs based on the same SNP data to identify potential loci under selection. For this data, we will subset just the northernmost and southernmost populations.

data<- read.vcfR("./filtered_3699snps_californicus.vcf")

## Scanning file to determine attributes.
## File attributes:
## meta lines: 9
## header_line: 10
## variant count: 3699
## column count: 726
## Meta line 9 read in.
## All meta lines processed.
## gt matrix initialized.
## Character matrix gt created.
## Character matrix gt rows: 3699
## Character matrix gt cols: 726
## skip: 0
## nrows: 3699
## row_num: 0
## Processed variant 1000Processed variant 2000Processed variant 3000Processed variant: 3699
## All variants processed

geno <- extract.gt(data)
dim(geno)

## [1] 3699 717

*#because the genotypes are not coded to what OutFLANK requires, we will edit the data a bit in here*
G <- geno
G[geno %in% c("0/0")] <- 0
G[geno %in% c("0/1")] <- 1
G[geno %in% c("1/1")] <- 2
G[is.na(G)] <- 9
tG <- t(G)
dim(tG)

## [1] 717 3699

subpops <- c("TBL","AK4")
subgen <- tG[meta$SITE%in%subpops,] *#subset method 1*

submeta <- subset(meta,SITE%in%subpops) *#subset method 2*
fst <- MakeDiploidFSTMat(subgen,locusNames=1:ncol(subgen),popNames=submeta$SITE)

## Calculating FSTs, may take a few minutes...

hist(fst$FST,breaks=50)


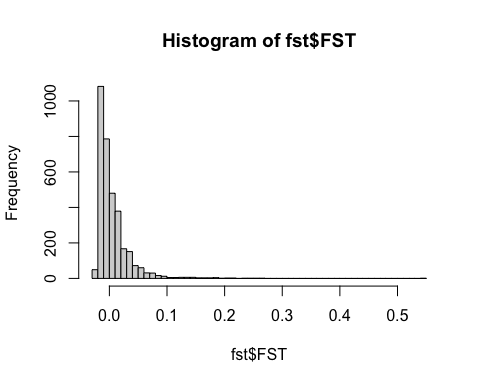


OF <- OutFLANK(fst,LeftTrimFraction=0.01,RightTrimFraction=0.01,
 Hmin=0.05,NumberOfSamples=2,qthreshold=0.01)
OutFLANKResultsPlotter(OF,withOutliers=T,
 NoCorr=T,Hmin=0.1,binwidth=0.005,
 Zoom=F,RightZoomFraction=0.05,titletext=NULL)


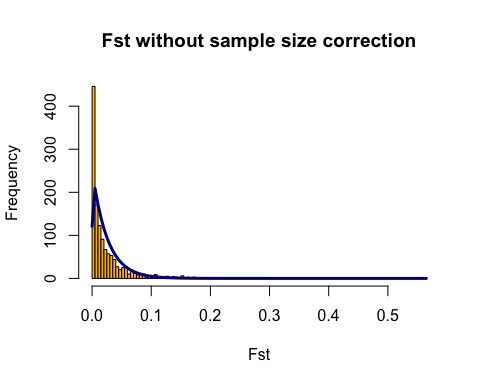
 Let’s look at FST outliers

P1 <- pOutlierFinderChiSqNoCorr(fst,Fstbar=OF$FSTNoCorrbar,
 dfInferred=OF$dfInferred,qthreshold=0.05,Hmin=0.1)
outliers <- P1$OutlierFlag==TRUE *#which of the SNPs are outliers?*
table(outliers)

## outliers
## FALSE TRUE
## 1273 17

plot(P1$LocusName,P1$FST,xlab="Position",ylab="FST",col=rgb(0,0,0,alpha=0.1))
points(P1$LocusName[outliers],P1$FST[outliers],col="magenta")


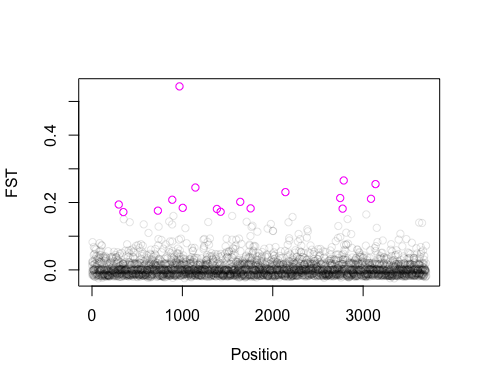


Ok, so using this subset of data, instead of 143 outlier SNPs based on PCA, we have 17 outlier SNPs based on FST.
